# Exploring thoracic aorta ECM alterations in Marfan syndrome: insights into aorta wall structure

**DOI:** 10.1038/s41598-025-09665-w

**Published:** 2025-07-22

**Authors:** Rodrigo Barbosa de Souza, Luara Lucena Cassiano, Philipp Barnowski, Sara Ventura, Walter Miguel Turato, Suelen Cristina Russafa Nascimento, Giovanna Lodi Mignanelli, Waldir Caldeira, Ana Maria Cristina Rebelo Pinto da Fonseca Martins, Flavio de Carvalho Luposeli, Francisco Rafael Martins Laurindo, Dieter P. Reinhardt, Gerhard Sengle, Ivan Hong Jun Koh, Keith M. Meek, Philip N. Lewis

**Affiliations:** 1 Department of Descriptive and Topographic Anatomy, Faculty of Santa Marcelina, São Paulo, Street Santa Marcelina, 91, 08270-140 SP Brazil; 2 Department of Genetics and Evolutionary Biology, University of São Paulo, São Paulo, 05508-090 SP Brazil; 3 Aquaculture Research and Development Center (CPDA), Fishing Institute of São Paulo, São Paulo, 04014-002 SP Brazil; 4 Department of Pediatrics and Adolescent Medicine, Faculty of Medicine and University Hospital Cologne, University of Cologne, 50931 Cologne, Germany; 5 Center for Biochemistry, Faculty of Medicine and University Hospital Cologne, University of Cologne, Cologne, Germany; 6 Vascular Biology Laboratory, The Heart Institute, University of Sao Paulo School of Medicine, São Paulo, 05403-000 SP Brazil; 7 Department of Clinical and Toxicological Analysis, University of São Paulo, São Paulo, 05508-000 SP Brazil; 8 Laboratory of Genetics and Molecular Cardiology, The Heart Institute, University of Sao Paulo School of Medicine, São Paulo, 05403-000 SP Brazil; 9 Department of Animal Sanity, Biologic Institute of São Paulo, São Paulo, 04014-002 SP Brazil; 10 Department of Surgery, Federal University of São Paulo, São Paulo, 04039-032 SP Brazil; 11 Faculty of Medicine and Health Sciences, McGill University, Montreal, QC Canada; 12 Faculty of Dentistry and Oral Health Sciences, McGill University, Montreal, QC Canada; 13 Center for Molecular Medicine Cologne (CMMC), University of Cologne, Cologne, Germany; 14 Cologne Center for Musculoskeletal Biomechanics (CCMB), 50931 Cologne, Germany; 15 Structural Biophysics Research Group, School of Optometry and Vision Sciences, Cardiff University, Maindy Road, Cathays, Cardiff, CF24 4HQ UK

**Keywords:** Marfan syndrome, Aorta, Extracellular matrix, Fibrillin-1, Aneurysm, Collagen, Cardiovascular genetics, Disease genetics, Anatomy, Aortic diseases, Experimental models of disease

## Abstract

Marfan syndrome is a connective tissue disorder caused by FBN1 mutations, leading to aortic wall fragility and increased susceptibility to aneurysm and dissection. This study investigated microstructural and molecular alterations in the thoracic aorta of Fbn1mgΔ^lpn^ mice, with a focus on the tunica intima and media. Histological and ultrastructural analyses demonstrated elastic fiber fragmentation and reduced fibrillin-1 expression. In the intima, endothelial cells showed partial detachment and decreased levels of fibrillin-1, perlecan, collagen IV, and α5β1 integrins, suggesting compromised adhesion to the extracellular matrix. Serial block-face scanning electron microscopy revealed discontinuities in the internal elastic lamina. In the media, we observed reduced fibronectin, altered α5β1 integrin distribution, and increased α-smooth muscle actin, indicative of remodeling in elastin–contractile units. Second harmonic generation imaging revealed increased collagen deposition, and thickness in areas of elastic fiber disruption, along with reduced and disorganized type III collagen and increased type I collagen. Echocardiographic evaluation showed aortic root, and ascendant-aorta dilatation, altered blood flow, and diastolic dysfunction. Elastic fiber integrity correlated strongly with fibrillin-1 expression (*r* = 0.93, *p* = 0.0003) and aortic blood flow (*r* = 0.77, *p* = 0.0064). These results suggest that early alterations in matrix organization and endothelial–matrix interactions may contribute to aortic wall weakening in Fbn1mgΔ^lpn^ mice.

**Supplementary Information:**

The online version contains supplementary material available at 10.1038/s41598-025-09665-w.

## Introduction

Marfan syndrome (OMIM #154700) (MFS) is a genetic disease characterized by mutations in the FBN1 gene^1,2^. The FBN1 gene induces alterations in the fibrillin-1 protein, which plays a crucial role in elastic fibers under normal physiological conditions^3^. Consequently, in Marfan syndrome (MFS), mutations in the FBN1 gene result in structural and functional alterations of the fibrillin-1 protein, leading to disrupted elastic fiber architecture^4^.

Under physiological conditions, the elastic fibers are composed of two main components: a central amorphous elastin core and a surrounding network of microfibrils^1,5^. Since the observations of Ross^6^, mature elastic fibers have been described as central amorphous region enclosed by a mesh of microfibrils. However, despite decades of study, several stages of elastic fiber assembly remain incompletely understood, particularly the phase in which elastin deposition depends on microfibrils to form mature fibers^7^.

Microfibril formation requires several key extracellular matrix components, including fibrillin-1, heparan sulfate, fibronectin, and integrins (α5β1, αvβ3, αvβ6). The deposition of the amorphous elastin core onto the microfibril scaffold involves proteins such as tropoelastin, fibrillins-4 and -5, and lysyl oxidase (LOX), which is essential for elastin cross-linking and maturation^8^.

Fibrillin-1 plays a central role by interacting with both heparan sulfate and integrins. Its N-terminal domain binds strongly to heparan sulfate, promoting microfibril assembly^9^, while the integrin-binding RGD (arginine-glycine-aspartic acid) motif mediates cell–matrix interactions through transmembrane integrin receptors^10,11^.

Fibronectin is also essential for the initial organization of the microfibrillar network and the formation of mature elastic fibers^12–14^. The long-term stability and maintenance of elastic fibers depend on several accessory proteins, including microfibril-associated proteins (MFAPs) such as MFAP4, which localizes to the interface between microfibrils and elastin^15^.

The incorporation of tropoelastin (the soluble precursor of elastin) into microfibrils involves multiple regulatory proteins^5,7^. LOX-mediated cross-linking of tropoelastin establishes permanent inter- and intramolecular bonds, leading to the formation of stable, mature elastin^16,17^.

Due to the widespread distribution of fibrillin-1, MFS manifests alterations in various organs^18^. However, common features of MFS include alterations in the cardiovascular system, skeleton, and eyes. Notably, the cardiovascular system receives significant attention due to premature death, often associated with aortic aneurysm rupture and aortic dissection^2,19^.

The structure of the aortic wall is divided into the tunica intima, media, and adventitia^20,21^. Both the tunica intima and tunica media contain elastic fibers in their wall structure^1,21^. Elastic fibers are essential components of the aorta, providing elasticity and allowing for distension and recoil during the cardiac cycle, the properties that are crucial for maintaining vascular homeostasis and normal mechanical function^22^.

In addition to fibrillin-1’s role in elastic fiber formation, it has been described to interact with other ECM components, such as perlecan^8^. Specifically, perlecan is a component of the basement membrane^23^, contributing to endothelial cell adhesion^24^. Besides the basement membrane, fibrillin-1 also supports endothelial adhesion through integrins, such as α5β1^25,26^.

In the tunica media, fibrillin-1 is involved in the elastic-contractile unit, promoting the maintenance of aortic biomechanics. This unit consists of interactions among elastin, fibrillin-1, integrins, and vascular smooth muscle (VSM) actin and myosin filaments, forming a dynamic link between the extracellular matrix and the contractile machinery of the vascular wall, due to the mechanical stresses applied to the aorta by the pulsatile blood flow^2,27–29^.

Various studies of the ECM in MFS have been carried out with the aim of improving clinical management. Animal models are commonly used to investigate the intrinsic mechanisms of the disease^30–35^.

The mgΔ^loxPneo^ (Fbn1 mgΔ^lpn^ mice) mouse is a dominant-negative model for Marfan syndrome. In this model, a 6-kb region of the Fbn1 gene encompassing exons 19–24 was replaced by a neomycin resistance cassette (neo), flanked by loxP sites, resulting in the deletion of 272 amino acid residues^36^. In the heterozygous state, mgΔ^lpn^ mice exhibit the classical Marfan phenotype, with alterations in the cardiovascular system, skeleton, lungs, and eyes^36,37^. Additionally, these animals exhibit a high incidence of aneurysms and aortic dissections^38^. Due to the similarities between the features of Fbn1 mgΔ^lpn^ mice and the MFS patient phenotype, this study aims to explore the ECM components of the tunica intima and media in the Fbn1 mgΔ^lpn^ mice to enhance understanding of the structure of the aorta’s wall in MFS.

## Results

### Structural organization of the aorta in MFS and WT groups

**Fig. 1:**
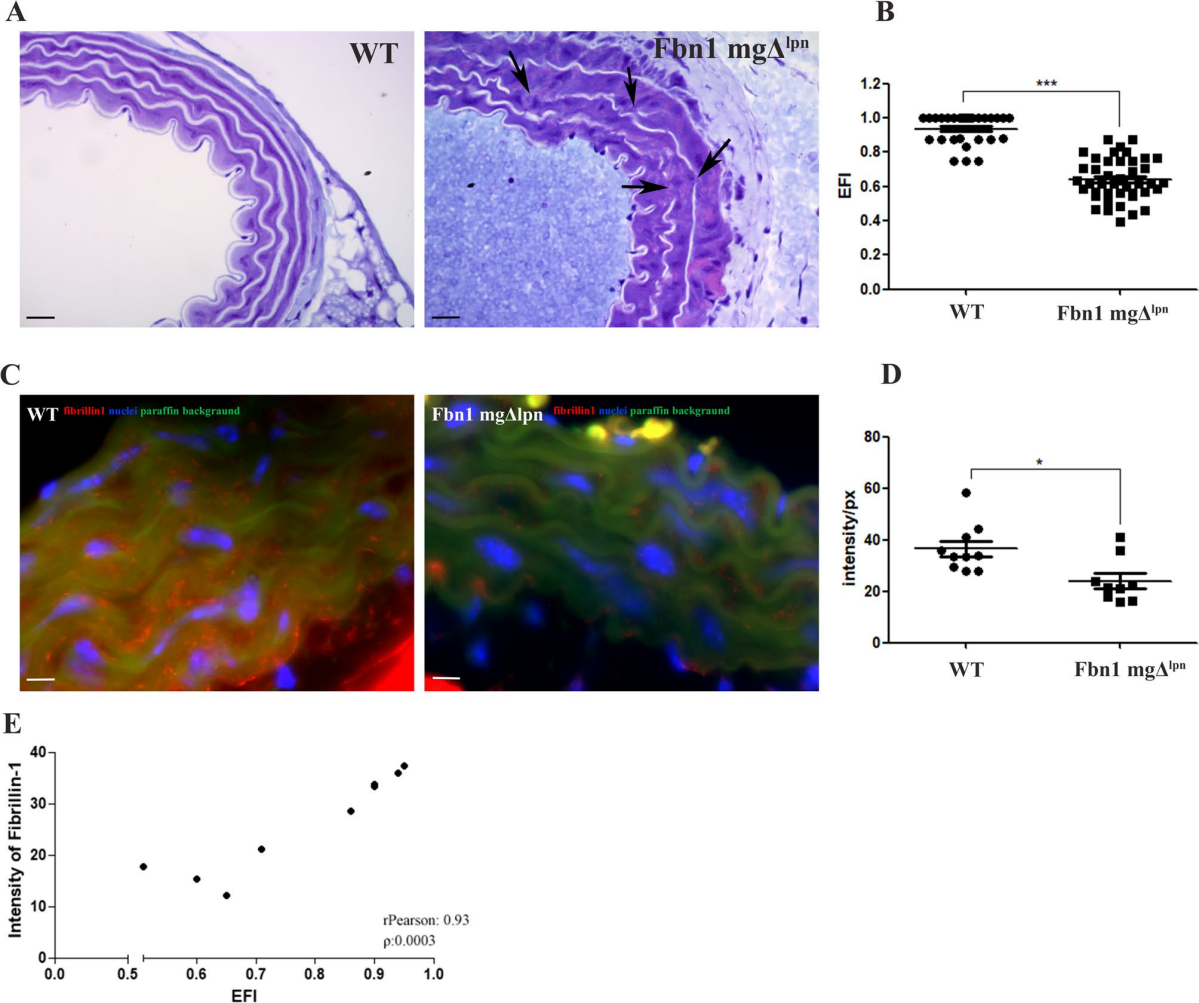
Histology and immunohistochemistry of the aorta (**A**). Representative images of aortic transverse sections stained with Toluidine Blue from both the WT group and the Fbn1 mgΔ^lpn^ mice group showed concentric elastic lamellae in white. In the Fbn1 mgΔ^lpn^ mice group tunica media fragmentation of the elastic fibers was observed (black arrows). Elastic Fiber Index (EFI) (**B**) showed a significant decrease of the elastic fiber integrity in the Fbn1 mgΔ^lpn^ mice group (0.63 ± 0.12) when compared to the WT group (0.93 ± 0.08). Fibrillin-1 immunofluorescence (**C**), showed the fibrillin-1 (red), the nuclei (blue), and the paraffin background (green). This indicated a reduction of fibrillin-1 distribution around of the elastic fibers in the Fbn1 mgΔ^lpn^ mice group, as well as a significant reduction of the fibrillin-1 intensity (WT 36.59_intensity/px_± 9.34_intensity/px_; MFS 24.06_intensity/px_± 8.79_intensity/px_) (**D**). **E**. Strong positive correlation between fibrillin-1 signal intensity and elastic fiber integrity (*r* = 0.93, *p* = 0.0003). (***) ρ < 0.001, and (*) ρ < 0.05; bar in A 10 μm, and in C 5 μm (Histological analysis WT *n* = 10; mgΔ^lpn^ *n* = 10, Fibrillin-1 immunofluorescence analysis: WT *n* = 10; Fbn1 mgΔ^lpn^ mice *n* = 9).

MFS is associated with elastic fiber fragmentation in the thoracic aorta^39^. In this study, the Fbn1 mgΔ^lpn^ mice group (0.63 ± 0.12) showed a significant reduction in elastic fiber integrity compared to the WT group (0.93 ± 0.08), as measured by EFI (Fig. 1A, B).

The principal structural components of the elastic fiber are tropoelastin and fibrillin^8^. As fibrillin-1 is known to be significantly modified in MFS^2^, the distribution of fibrillin-1 was examined. A significant reduction in fibrillin-1 was observed in the Fbn1 mgΔ^lpn^ mice group (24.06_intensity/px_± 8.79_intensity/px_) compared to the WT group (36.59_intensity/px_± 9.34_intensity/px_) (Fig. 1C and D). Interestingly, a reduction in fibrillin-1 immunolocalization was observed along the concentric elastic lamellae in the tunica media of the Fbn1 mgΔ^lpn^ mice group. The interlamellar spaces showed very low fibrillin-1 signal in both groups. Furthermore, we found a strong positive correlation (Fig. 1E) between fibrillin-1 signal intensity and elastic fiber integrity (*r* = 0.93, *p* = 0.0003), suggesting that reduced fibrillin-1 may contribute to impaired organization of elastic structures in the aortic wall.

### Tunica intima in the MFS

**Fig. 2:**
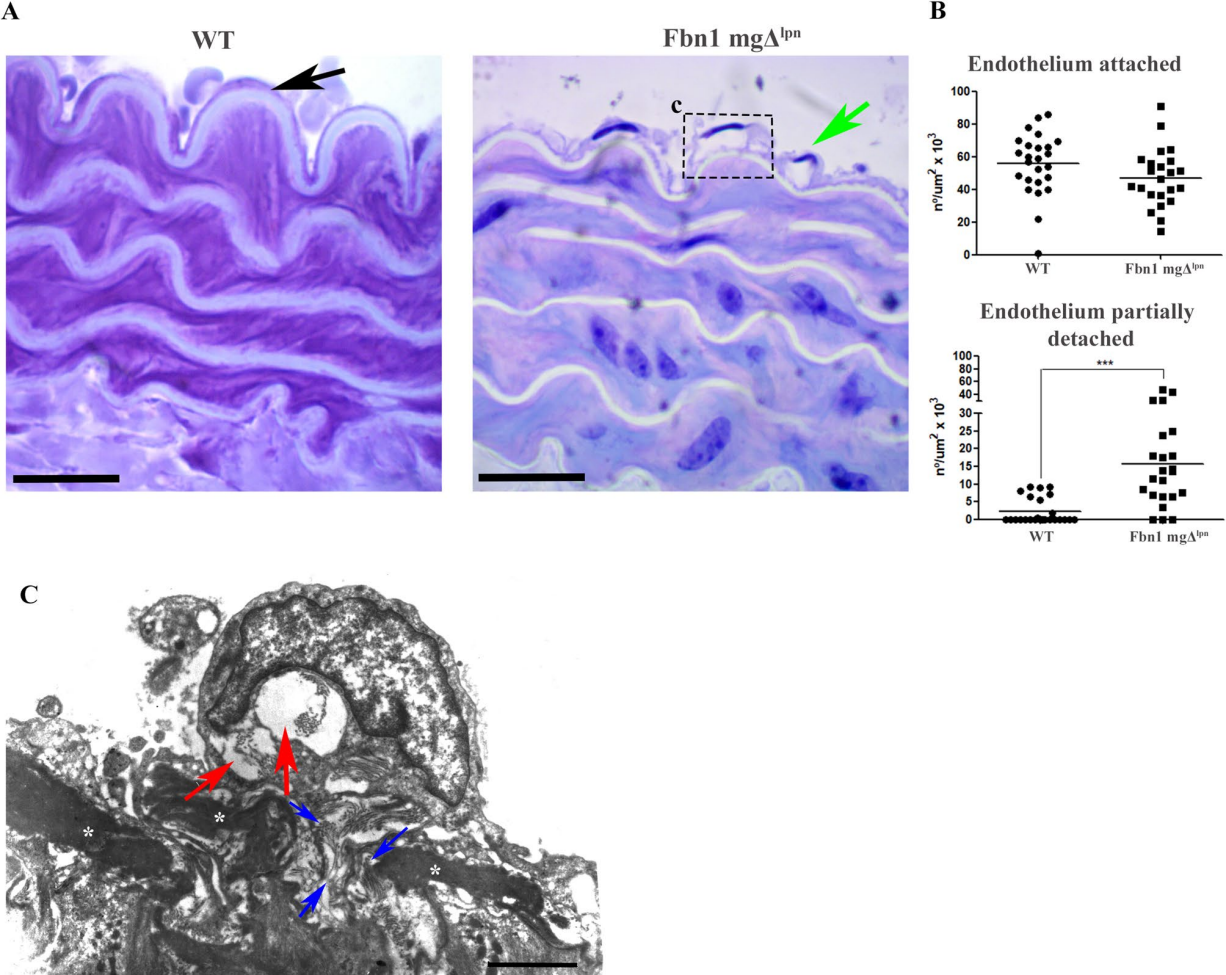
Histological analysis of the tunica intima (**A**, **B**). In the WT group (**A**), endothelial cells were observed resting on the tunica intima with a flat nucleus, characteristic of squamous epithelium (black arrow). In contrast, the Fbn1 mgΔ^lpn^ mice group (**B**) exhibited partially detached endothelial cells (green arrow). No significant difference was observed in the Endothelium Attached Index between groups (WT: 55.60 ± 19.01; Fbn1 mgΔ^lpn^ mice: 47.00 ± 17.77). However, the Endothelium Detached Index was significantly higher in the Fbn1 mgΔ^lpn^ mice group (WT: 2.25 ± 3.59; Fbn1 mgΔ^lpn^ mice: 15.69 ± 13.22). Transmission electron microscopy (TEM) analysis **C**. TEM images highlighted the presence of partially detached endothelial cells (red arrows) in the Fbn1 mgΔ^lpn^ mice group (**C**). Additionally, fragmentation of elastic fibers (*) was observed, along with extracellular matrix fibers located in regions of elastic fiber fragmentation (blue arrows) and endothelial cells exhibiting a more rounded morphology than normal. The white asterisk (*) indicates elastic fibers; (***) ρ < 0.001. Scale bars: 5 μm for A and B; 1900 nm for C. (Histological analysis: WT *n* = 10; Fbn1 mgΔ^lpn^ mice *n* = 10, TEM analysis Fbn1 mgΔ^lpn^ mice *n* = 3)

The tunica intima has been described as a layer composed of endothelial cells on a basement membrane, which overlays an internal elastic lamina (IEL)^21^. Histologically, in the WT group, endothelial cells were observed resting on the tunica intima with a flat nucleus. However, in the Fbn1 mgΔ^lpn^ mice group, some endothelial cells appeared partially detached (Fig. 2A). Quantitative analysis of the number of cells per unit area revealed a significantly higher Endothelium Detached Index in the Fbn1 mgΔ^lpn^ mice group (15.69 ± 13.22) compared to the WT group (2.25 ± 3.59) (Fig. 2B). Interestingly, no significant differences were found between the Fbn1 mgΔ^lpn^ mice (47.00 ± 17.77) and WT (55.60 ± 19.01) groups in the number of attached endothelial cells (Fig. 2B). Due to the partially detached appearance of endothelial cells in the Fbn1 mgΔ^lpn^ mice group, TEM analysis was performed for further investigation. This analysis revealed the fragmentation of elastic fibers, extracellular matrix fibers in regions of elastic fiber fragmentation, and endothelial cells with a more rounded morphology than normal (Fig. 2C).

**Fig. 3:**
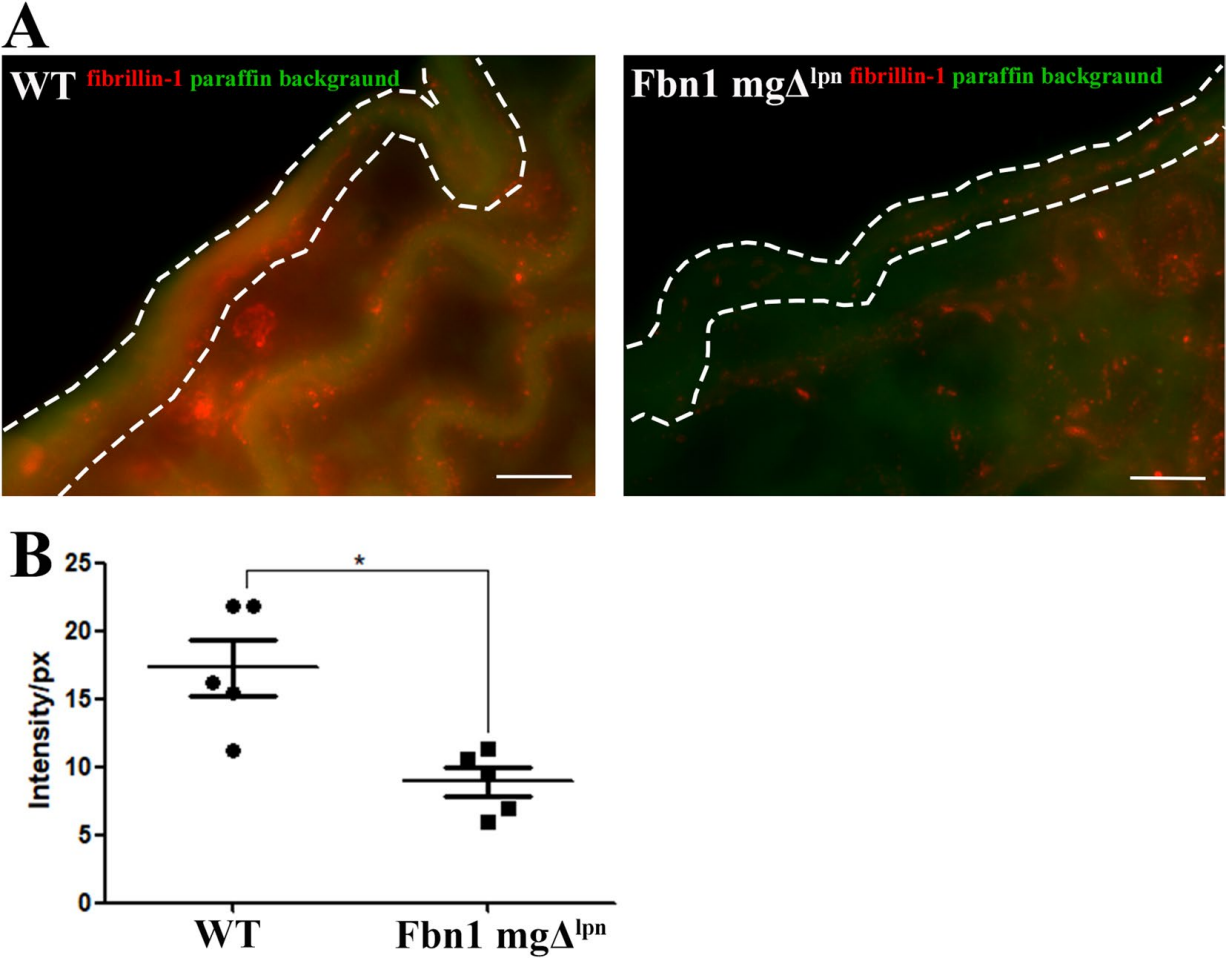
Fibrillin-1 immunofluorescence analysis in the Fbn1 mgΔ^lpn^ mice group revealed a reduction in fibrillin-1 distribution around the elastic fibers in tunica intima (white dotted line) (**A**). The intensity of fibrillin-1 staining in the tunica intima was significantly lower in the Fbn1 mgΔ^lpn^ mice group (8.90_intensity/px_± 2.3_intensity/px_) compared to the WT group (17.29_intensity/px_± 4.5_intensity/px_). (*) ρ < 0.05. Scale bar: 5 μm (**A**). (Fibrillin-1 immunofluorescence analysis: WT *n* = 5; Fbn1 mgΔ^lpn^ mice *n* = 5)

**Fig. 4:**
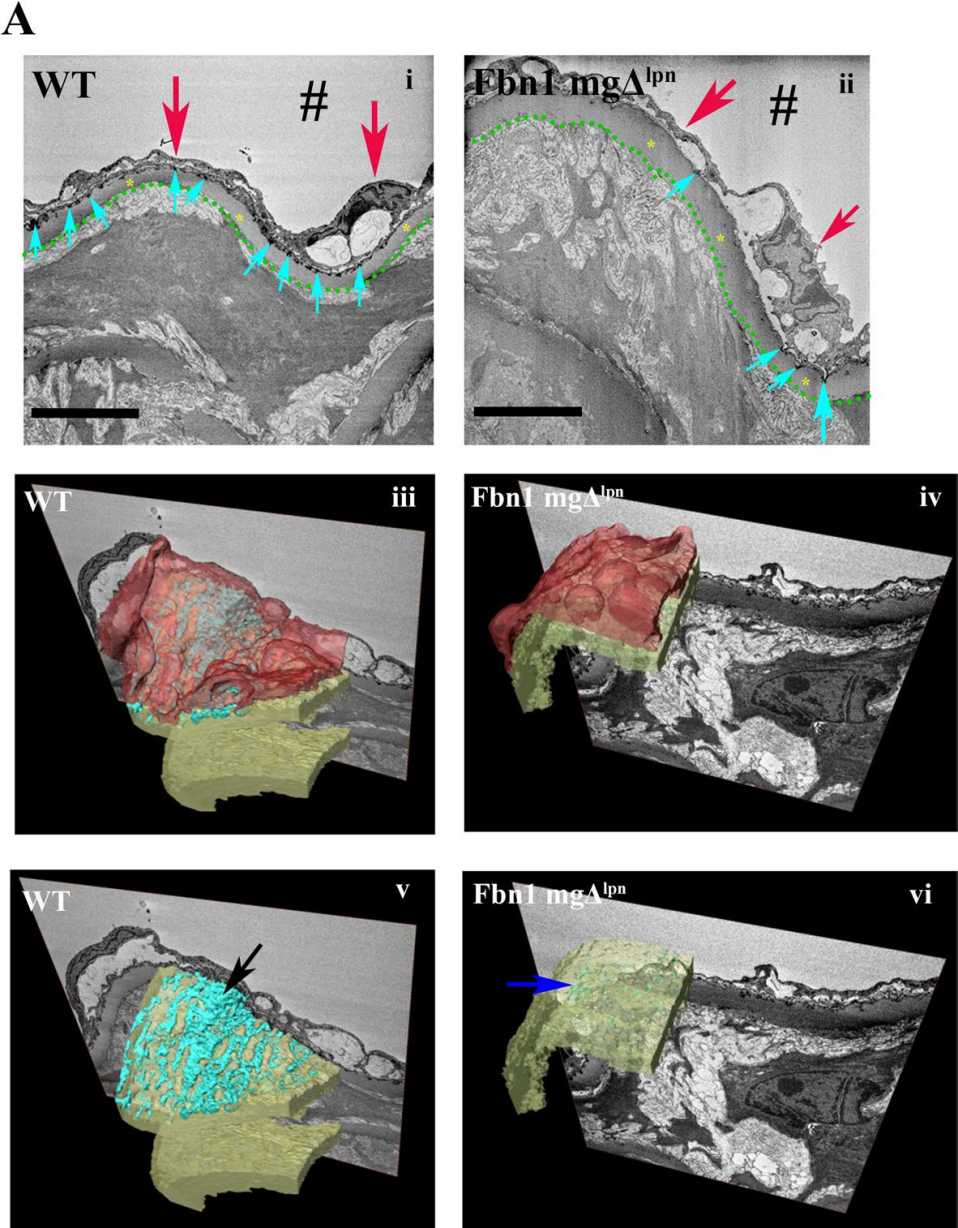
3D reconstruction of the internal elastic lamina (IEL). **A** Representative 2D images prior to reconstruction from the WT group (i) and the Fbn1 mgΔ^lpn^ mice group (ii). The dotted green line delineates the boundary between the tunica intima and the tunica media. Yellow asterisks indicate elastic lamellae, light blue arrows highlight the IEL, characterized by electron-dense fibers, and red arrows mark endothelial cells. In the 3D reconstructions (WT iii and v; Fbn1 mgΔlpn mice iv and vi), endothelial cells are shown in red, the IEL in light blue, and the remaining elastic lamellae in yellow. In the WT group (v), the IEL forms a well-organized tubular network (black arrow), whereas in the Fbn1 mgΔlpn mice group, a reduction of the IEL network is observed (blue arrow). The “#” indicate the aorta lumen, scale bar: 5 μm. Sample size: WT *n* = 2; Fbn1 mgΔ^lpn^ mice *n* = 2.

Mariko et al.^40^ described that fibrillin-1 mediates adhesion of endothelial cells. We analyzed the fibrillin-1 in the tunica intima by immunofluorescence. The intensity of fibrillin-1 staining was significantly higher in the WT group (17.29_intensity/px_± 4.5_intensity/px_) compared to the Fbn1 mgΔ^lpn^ mice group (8.90_intensity/px_± 2.3_intensity/px_), where its presence was reduced in the tunica intima (Fig. 3A, B). Fibrillin-1 is an essential protein for the formation of microfibrils and elastic fibers. To further investigate alterations in the distribution of microfibrils within the tunica intima, serial block-face scanning electron microscopy (SBF-SEM) was employed for three-dimensional analysis. In the WT group, the internal elastic lamina (IEL), which is the innermost elastic lamella directly adjacent to the endothelium, formed a sheet-like fenestrated network of interconnected elastic fibers. Beneath the IEL, additional elastic lamellae are organized in layers contributing to the vessel wall’s elastic structure (Fig. 4Ai, Aiii, Av, and Supplementary video 1). In contrast, the Fbn1 mgΔ^lpn^ mice exhibited a disrupted IEL network that had lost its sheet-like integrity and appeared fragmented within the elastic lamellae (Fig. 4Aii, Aiv, Avi, and Supplementary video 2).

**Fig. 5:**
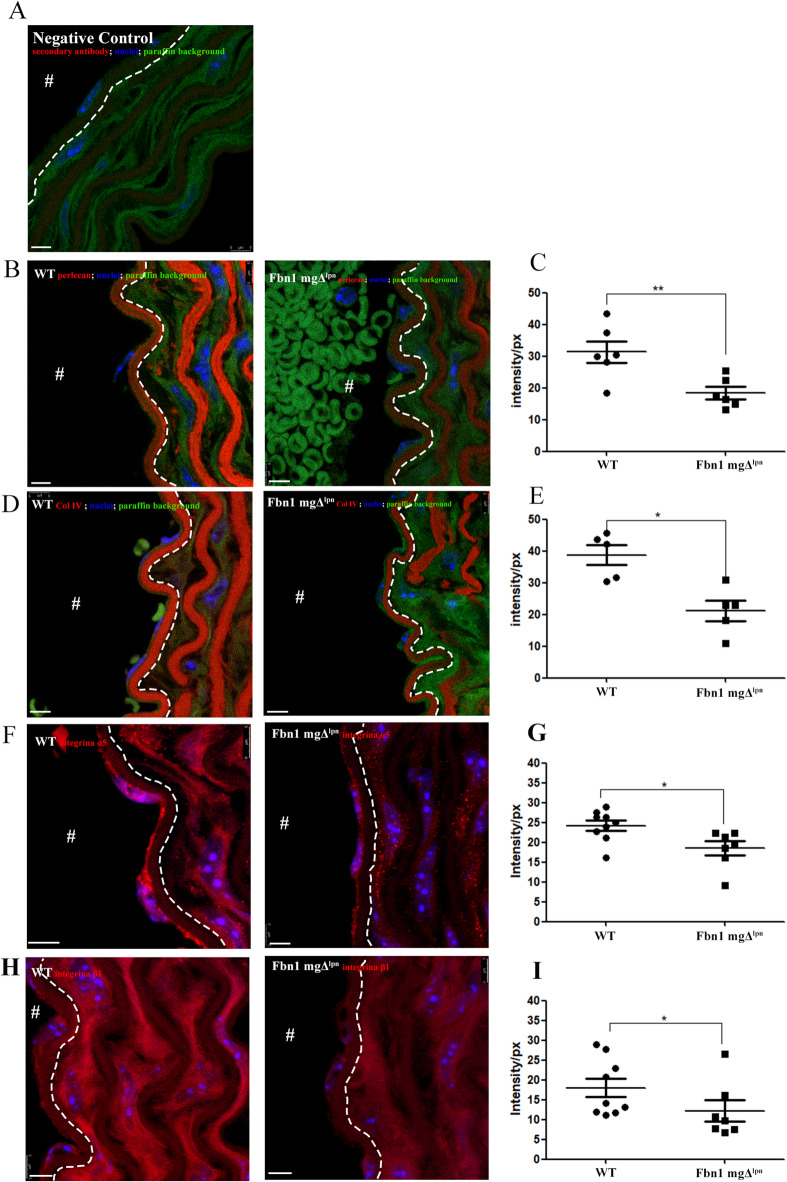
Basement membrane and tunica intima. Negative control (**A**) showed the secondary antibody (red), nuclei (blue), and green (green). Perlecan immunofluorescence (**B**) showed the perlecan (red), and alongside the nuclei (blue). The Fbn1 mgΔ^lpn^ mice group displayed a significant reduction of the perlecan intensity (WT 31.32_intensity/px_± 8.45_intensity/px_; Fbn1 mgΔ^lpn^ mice 18.38_intensity/px_± 4.68_intensity/px_) (**C**). Collagen type IV immunofluorescence (**D**), revealed the collagen type IV (red), and alongside the nuclei (blue). The Fbn1 mgΔ^lpn^ mice group showed a significant reduction of the collagen type IV intensity (WT 38.72_intensity/px_± 7.08_intensity/px_; Fbn1 mgΔ^lpn^ mice 21.19_intensity/px_± 7.30_intensity/px_) (**E**). Integrin α5 immunofluorescence (**F**), showed the integrin α5 (red), and alongside the nuclei (blue), The Fbn1 mgΔ^lpn^ mice group showed a significant reduction of the integrin α5 intensity (WT 24.21_intensity/px_± 3.87_intensity/px_; Fbn1 mgΔ^lpn^ mice 18.50_intensity/px_± 4.65_intensity/px_) (**G**). Integrin β1 immunofluorescence (**H**), showed the integrin β1 (red), and alongside the nuclei (blue)The Fbn1 mgΔ^lpn^ mice group showed a significant decrease of the integrin β1 (WT 18.02_intensity/px_± 7.14_intensity/px_; Fbn1 mgΔ^lpn^ mice 12.20_intensity/px_± 7.03_intensity/px_) (**I**). dotted white line separed the tunica intima and tunica media; The “#” white indicate the aorta lumen; (**) ρ < 0.001, and (*) ρ < 0.05; bar in A, B, D, F, and H 5 μm (immunofluorescence analysis: Perlecan WT *n* = 6; Fbn1 mgΔ^lpn^ mice *n* = 6, Collagen type IV WT *n* = 5; Fbn1 mgΔ^lpn^ mice *n* = 5, Integrin α5 and β1WT *n* = 9; Fbn1 mgΔ^lpn^ mice *n* = 7).

Interestingly, Tiedemann et al.^41^ reported that fibrillin-1 co-localizes with perlecan in various basement membranes (BM). Perlecan is known to play a critical role in cell adhesion^42^. Similar to the findings of Nonaka et al.^43^, we observed the distribution of perlecan around the elastic fibers in the WT group. However, in the Fbn1 mgΔ^lpn^ mice group, there was a notable reduction in perlecan signal intensity. Quantitative analysis revealed a significant decrease in perlecan intensity in the MFS group (18.38_intensity/px_± 4.68_intensity/px_) compared to the WT group (31.32_intensity/px_± 8.45_intensity/px_) (Fig. 5B and C). Another component of the basement membrane (BM) is collagen type IV^23^. In this study, collagen type IV was observed to be distributed around the elastic fibers and between cells and elastic fibers in the WT group, a localization pattern similar with previous findings^44,45^. In contrast, the Fbn1 mgΔ^lpn^ mice group showed a significant reduction in collagen type IV signal intensity (21.19_intensity/px_± 7.30_intensity/px_) compared to the WT group (38.72_intensity/px_± 7.08_intensity/px_) (Fig. 5D and E).

Cell interactions with the ECM are also known to occur through binding integrin by RGD (Arg-Gly-Asp) sites^46^. Both collagen type IV and fibrillin-1 have this site and interact with α5β1 integrin^8,47^. This study observed a significant decrease in integrin α5 in the Fbn1 mgΔ^lpn^ mice group (18.50_intensity/px_± 4.65_intensity/px_), in contrast to the WT group (24.21_intensity/px_± 3.87_intensity/px_) and a significant reduction of the integrin β1in Fbn1 mgΔ^lpn^ mice group (WT 18.02_intensity/px_± 7.14_intensity/px_; Fbn1 mgΔ^lpn^ mice 12.20_intensity/px_± 7.03_intensity/px_) (Fig. 5F–I).

The impairment of binding integrity between endothelial cells observed in the Fbn1 mgΔ^lpn^ mice group can be likely related to the reduction of fibrillin-1 and the consequent loss of key cell adhesion molecules with which it interacts, such as perlecan. The decreased expression of integrin α5 and integrin β1 may also contribute to alterations in the tunica intima. Changes in fibrillin-1 expression can induce multifactorial modifications in the tunica intima, can potentially compromising endothelial cell adhesion.

### Tunica media in MFS model

The tunica media is a layer that includes several elastic lamellae organized concentrically. Between elastic lamellae of the tunica media comprises vascular smooth muscle cells (VSMC), collagen fibers, proteoglycans, and glycoproteins^7^.

**Fig. 6:**
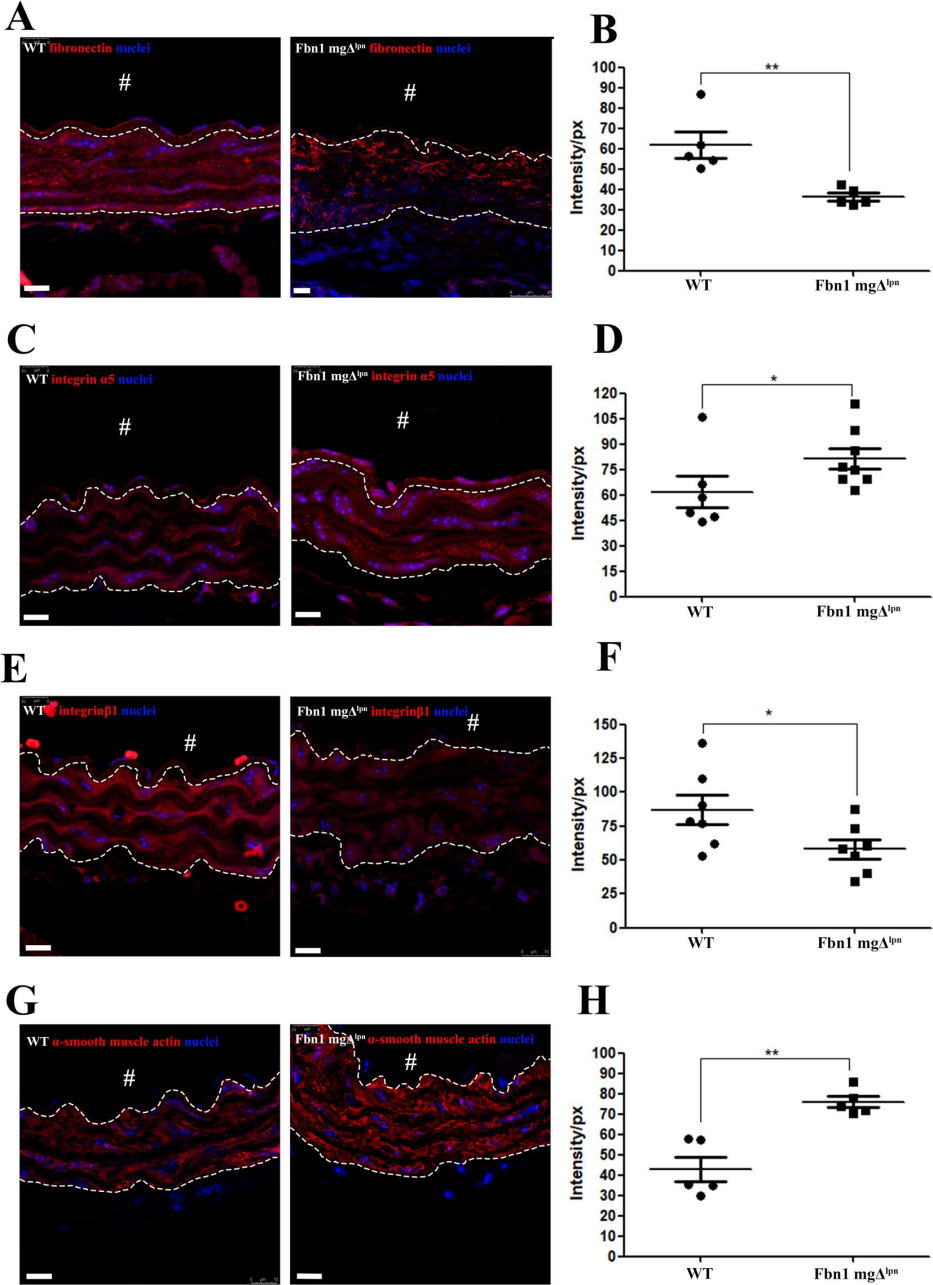
Thoracic aorta tunica media. Fibronectin immunofluorescence (**A**), showed the fibronectin (red), and the nuclei (blue). The Fbn1 mgΔ^lpn^ mice group showed a significant reduction of the fibronectin intensity (61.91_intensity/px_± 14.47_intensity/px_; Fbn1 mgΔ^lpn^ mice 36.00_intensity/px_± 4.27_intensity/px_) (**B**). Integrin α5 immunofluorescence (**C**), showed the Integrin α5 (red), and the nuclei (blue). The Fbn1 mgΔ^lpn^ mice group showed a significant increase in the integrin α5 intensity (WT 61.89_intensity/px_± 23.06_intensity/px_; Fbn1 mgΔ^lpn^ mice 81.39_intensity/px_± 17.06_intensity/px_) (**D**). Integrin β1 immunofluorescence (**E**), showed the integrin β1 (red), and the nuclei (blue). The Fbn1 mgΔ^lpn^ mice group showed a significant reduction of the integrin β1 intensity (WT 86.82_intensity/px_± 28.73_intensity/px_; Fbn1 mgΔ^lpn^ mice 57.99_intensity/px_± 18.18_intensity/px_) (**F**). α-smooth muscle actin immunofluorescence (**G**), showed the α-smooth muscle actin (red), and the nuclei (blue). The Fbn1 mgΔ^lpn^ mice group showed a significant increase in the α-smooth muscle actin intensity (WT 42.97_intensity/px_± 13.46_intensity/px_; Fbn1 mgΔ^lpn^ mice 75.97_intensity/px_± 6.26_intensity/px_) (**H**).The “#” white indicate the aorta lumen; (**) ρ < 0.005, and (*) ρ < 0.05; bar in A, C, E, and G 5 μm, space between dotted white lines is the tunica media. (immunofluorescence analysis: Fibronectin WT *n* = 5; Fbn1 mgΔ^lpn^ mice *n* = 5, Integrin α5 WT *n* = 6; Fbn1 mgΔ^lpn^ mice *n* = 8; Integrin β1WT *n* = 7; Fbn1 mgΔ^lpn^ mice *n* = 7, α-smooth muscle actin WT *n* = 5; Fbn1 mgΔ^lpn^ mice *n* = 5).

Fibrillin-1 is a pivotal fibrillar component of elastic fiber assembly, but fibronectin is also a critical prerequisite for assembly^8,48^. In the tunica media, a significant reduction of fibronectin was observed in the Fbn1 mgΔ^lpn^ mice group (36.00_intensity/px_± 4.27_intensity/px_) in comparison to the WT group (61.91_intensity/px_± 14.47_intensity/px_) (Fig. 6B). To clarify this finding, the tunica media was delineated with a dashed line in the representative images. In addition, the fibronectin distribution in the Fbn1 mgΔ^lpn^ mice group was reduced both in the interlamellar spaces and along the outer surfaces of the elastic lamellae when compared to the WT group. (Fig. 6A).

Due to interaction of the fibrillin-1 with integrins^2^, in this study, we investigated fibrillin-1 associated α5 integrin and β1 integrin expression to determine how the integrins expression is affected in the Fbn1 mgΔ^lpn^ mice model. The Fbn1 mgΔ^lpn^ mice group revealed a significant increase in the intensity of integrin α5 (81.39_intensity/px_± 17.06_intensity/px_), in contrast to the WT group (61.89_intensity/px_± 23.06_intensity/px_) (Fig. 6C, D). However, integrin β1 showed a significant reduction in the Fbn1 mgΔ^lpn^ mice (WT 86.82_intensity/px_± 28.73_intensity/px_; Fbn1 mgΔ^lpn^ mice 57.99_intensity/px_± 18.18_intensity/px_) (Fig. 6E, F).

In addition, this study also identified a significant increase in α-smooth muscle actin in the Fbn1 mgΔ^lpn^ mice group (75.97_intensity/px_± 6.26_intensity/px_), compared to the WT group (42.97_intensity/px_± 13.46_intensity/px_) (Fig. 6G and H). These findings indicate that components associated with the elastin-contractile unit exhibit altered expression in this model, in line with previous studies in Marfan syndrome and related aortopathies^2,27–29,49,50^.

Interestingly, analysis of the spectral curve of aortic blood flow in the Fbn1 mgΔ^lpn^ mice group revealed atypical figures, such as an inconsistently present systolic peak and a diastolic curve with peaks (Supplementary Fig. 1A). In contrast, the WT group exhibited a uniform peak in both systolic and diastolic curves (Supplementary Fig. 1A). Moreover, the Fbn1 mgΔ^lpn^ mice group demonstrated a significant reduction in blood flow in the upper abdominal aorta (WT 2.93mL/min ± 0.34mL/min, Fbn1 mgΔ^lpn^ mice 1.99mL/min ± 0.63mL/min) (Supplementary Fig. 1B). Blood flow was also positively correlated with elastic fiber integrity (Spearman *r* = 0.77, *p* = 0.0064) (Supplementary Fig. 1C). Echocardiography showed significant dilation of the aortic root (WT: 1.64 mm ± 0.10; Fbn1 mgΔlpn mice: 2.39 mm ± 0.59) and ascending aorta (WT: 1.60 mm ± 0.09; Fbn1 mgΔlpn mice: 3.40 mm ± 0.89) (Table 1, Supplementary Fig. 2A). While most cardiac parameters, including cardiac output and chamber volume, were unchanged and no valve dysfunction was observed. Early diastolic dysfunction was evident, with a significant reduction in E wave velocity (WT: 534.0 mm/s ± 123.3; Fbn1 mgΔlpn mice: 303.10 mm/s ± 48.42) and E/A ratio (WT: 2.43 ± 0.92; Fbn1 mgΔlpn mice: 0.94 ± 0.15) (Table 1), suggest impaired left ventricular relaxation. Given the absence of impaired ventricular systolic dysfunction and valve defects, we propose that impaired blood flow may be due to a perturbed distribution of cardiac output, with a larger proportion of blood being preferentially diverted to the more compliant conductance vessels of the upper body. In fact, the correlation between blood flow changes and degree of elastic fiber breaks is consistent with this possibility.

**Table 1:** Cardiac function measurements in Fbn1 mgΔ^lpn^ mice model for Marfan syndrome.

Cardiac Function Measurements	WT	Fbn1 mgΔ^lpn^ mice	ρ-value
A wave	251.80_mm/s_±115.20_mm/s_	323.50_mm/s_±46.80_mm/s_	0,4318
A’ wave	13.58_mm/s_±2.44_mm/s_	16.89_mm/s_±5.54_mm/s_	0,5303
**E wave**	**534.0**_**mm/s**_ **±123.3**_**mm/s**_	**303.10**_**mm/s**_ **±48.42**_**mm/s**_	**0**,**0092**
E’ wave	19.68_mm/s_±6.88_mm/s_	15.60_mm/s_±3.19_mm/s_	0,3434
Isovolumetric Contraction Time	19.30 ± 7.36	17.00 ± 5.16	0,5303
Isovolumetric Relaxation Time	23.82_ms_ ± 9.36_ms_	22.15_ms_ ± 5.48_ms_	0,7551
E wave deceleration	22.48_ms_ ± 6.66_ms_	19.07_ms_ ± 3.02_ms_	0,5691
Ejection time	45.58_ms_ ± 9.98_ms_	50.24_ms_ ± 8.88_ms_	0,4318
Ejection Fraction	29.94_%_±2.32_%_	32.15_%_±12.25_%_	0,9273
S’ wave	15.70_mm/s_±3.01_mm/s_	15.84_mm/s_±3.70_mm/s_	0,8763
A’/E’ ratio	0.77 ± 0.29	1.09 ± 0.30	0,0727
**E/A ratio**	**2.43 ± 0.92**	**0.94 ± 0.15**	**0**,**0025**
E/E’	28.49 ± 6.42	20.29 ± 6.10	0,1061
Myocardial Performance Index	1.02 ± 0.48	0.79 ± 0.18	0,6389
Heart Rate	407.8_BPM_ ± 57.73_BPM_	426.7_BPM_ ± 38.21_BPM_	0,5699
**Root Aortic**	**1.64**_**mm**_ **± 0.10**_**mm**_	**2.39**_**mm**_ **± 0.59**_**mm**_	**0**,**0051**
**Ascending Aorta**	**1.60**_**mm**_ **± 0.09**_**mm**_	**3.40**_**mm**_ **± 0.89**_**mm**_	**0**,**0025**

A significant reduction in the E wave and E/A ratio was observed in *Fbn1*mgΔ^lpn^ mice, indicating impaired left ventricular relaxation a hallmark of early diastolic dysfunction. Additionally, a significant increase in the diameters of the aortic root and ascending aorta was found in these animals. Differences were considered statistically significant at a ρ-value < 0.05. Data are expressed as mean ± standard deviation. (WT *n* = 5; Fbn1 mgΔ^lpn^ mice *n* = 5).

**Fig. 7:**
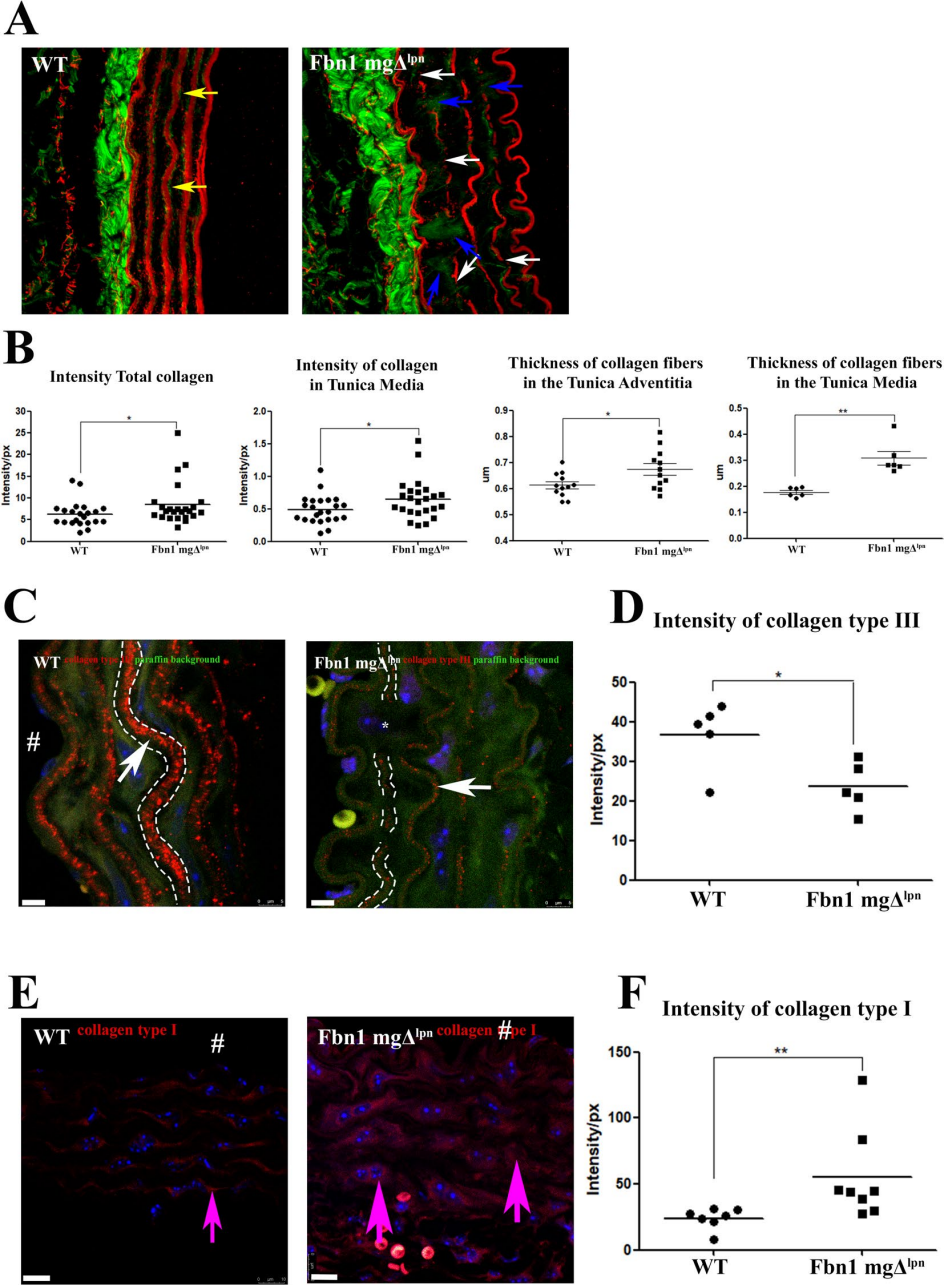
Collagen distribution in the aorta. **A** Collagen distribution by SHG (green) and tropoelastin (red); The WT group showed a uniform tropoelastin marker, and around tropoelastin the discrete presence of the collagen fibers (yellow arrows). Fbn1 mgΔ^lpn^ mice showed a discontinuous intensity of the tropoelastin marker (white arrows), and within the apparent fragmentation of elastic fibers, collagen deposition was observed (blue arrows). **B** The intensity of total collagen fibers (WT 6.3_intensity/px_± 2.9_intensity/px_; Fbn1 mgΔ^lpn^ mice 8.5_intensity/px_± 4.89_intensity/px_), and of collagen in the tunica media (WT 0.49_intensity/px_± 0.21_intensity/px_; Fbn1 mgΔ^lpn^ mice 0.64_intensity/px_± 0.30_intensity/px_) were both significantly increased in the Fbn1 mgΔ^lpn^ mice group. The thickness of collagen fibers in the tunica adventitia was observed the significantly increase in Fbn1 mgΔ^lpn^ mice group when compared to WT group (WT: 0.61 μm ± 0.04 μm; Fbn1 mgΔ^lpn^: 0.67 μm ± 0.07 μm). Besides, in the tunica media, a significant increase in collagen fiber thickness was also observed in Fbn1 mgΔ^lpn^ mice compared to WT (WT: 0.17 μm ± 0.01 μm; Fbn1 mgΔ^lpn^: 0.30 μm ± 0.064 μm). Collagen type III immunofluorescence (**C**), showed in red the collagen type III (white arrow), and in blue the nuclei. **D** The Fbn1 mgΔ^lpn^ mice group showed a significant reduction of the collagen type III intensity (WT 36.78_intensity/px_± 8.5_intensity/px_; Fbn1 mgΔ^lpn^ mice 23.57.57_intensity/px_± 6.2_intensity/px_). The distribution of collagen type III closely follows the contours of the elastic lamellae (dotted white line). In Fbn1 mgΔ^lpn^ mice, reduced collagen type III intensity and its absence in regions of elastic fiber fragmentation (*) were observed. Collagen type I immunofluorescence (**E**), showed the collagen type I (red – pink arrow), and the nuclei (blue). **F** The Fbn1 mgΔ^lpn^ mice group showed a significant increase of the collagen type I intensity (WT 24.01_intensity/px_± 7.79_intensity/px_; Fbn1 mgΔ^lpn^ mice 55.47_intensity/px_± 34.4_intensity/px_) (**F**). (*) ρ < 0.05 (***), ρ < 0.001; bar in **C** and **E** are 5 μm. The “ # ” indicates the aorta lumen (**C** and **E**), “ * ” indicates elastic fiber fragmentation; dotted white lines indicate elastic fibers. (SHG: WT *n* = 6; Fbn1 mgΔ^lpn^ mice *n* = 6, Collagen type III WT *n* = 5; Fbn1 mgΔ^lpn^ mice *n* = 5, Collagen type I WT *n* = 7; Fbn1 mgΔ^lpn^ mice *n* = 8).

Besides, in normal physiological conditions, aortic tissue is characterized by the presence of collagen types I and III^21^, with collagen type III exhibiting a more widespread distribution than type I^51^. Using SHG we observed a significant increase in both total collagen (WT 6.3_intensity/px_± 2.9_intensity/px_; MFS 8.5_intensity/px_± 4.89_intensity/px_) and tunica media collagen (WT 0.49_intensity/px_± 0.21_intensity/px_; MFS 0.64_intensity/px_± 0.30_intensity/px_), in the Fbn1 mgΔ^lpn^ mice group (Fig. 7A, B). Compared to WT mice, the Fbn1 mgΔ^lpn^ group exhibited a significantly greater collagen fiber diameter in the tunica adventitia (WT: 0.61 μm ± 0.04 μm; Fbn1 mgΔ^lpn^: 0.67 μm ± 0.07 μm) (Fig. 7B), which corresponds to the thickness of approximately one to two fibrils, depending on the collagen type^52^. It is important to acknowledge the limitations of this measurement approach. The mean diameter in both groups is approximately 640 nm, equivalent to ~ 3.4 pixels (7 px/µm), which hinders precise boundary definition.

In the tunica media, a significant increase in collagen fiber thickness was also observed in Fbn1 mgΔ^lpn^ mice compared to WT (WT: 0.17 μm ± 0.01 μm; Fbn1 mgΔ^lpn^: 0.30 μm ± 0.064 μm) (Fig. 7B). However, collagen fibers in this layer are extremely thin and appear in multiple orientations, both longitudinal and radial, which may reduce laser excitation efficiency and limit SHG signal detection compared to the adventitial region. Although we applied the thresholding tool in FIJI, as previously described^53^, to enhance collagen visualization, it is possible that the measurements also captured general SHG signal distribution within the tunica media.

These results led us to investigate the predominant collagen type distribution in Fbn1 mgΔ^lpn^ mice group. The Fbn1 mgΔ^lpn^ mice group displayed a significant reduction in collagen fiber type III in aortic tissue (WT 36.78_intensity/px_± 8.5_intensity/px_; Fbn1 mgΔ^lpn^ mice 23.57_intensity/px_± 6.2_intensity/px_), particularly in the region associated with elastic fiber fragmentation (Fig. 7C, D). Furthermore, the WT group exhibited the deposition of collagen fiber type III is predominantly within the interlamellar regions of the tunica media, and their distribution closely follows the contours of the elastic lamellae. In contrast, the Fbn1 mgΔ^lpn^ mice show a reduction in collagen type III signal within the interlamellar space and along the elastic lamellae contours, indicating a disrupted spatial distribution (Fig. 7C).

Analysis of collagen type I revealed a significant increase in the Fbn1 mgΔ^lpn^ mice group (WT 24.01_intensity/px_± 7.79_intensity/px_; MFS 55.47_intensity/px_± 34.4_intensity/px_) (Fig. 7E, F). Additionally, it was observed that collagen type I was localized around the VSCM between elastic fibers.

## Discussion

The cardinal manifestation of MFS is cardiovascular alterations, which are known to progressively worsen over time^1,19,54^. The symptoms of MFS are related to a mutation in the FBN1 gene^19^. In this study, we utilized the Fbn1 mgΔ^lpn^ mice model, a dominant-negative model that exhibits the classic phenotype of MFS disease, including cardiovascular, skeletal, and ocular alterations ^36–38,55^.

In the Fbn1 mgΔ^lpn^ mice group, we observed a significant reduction in the distribution and intensity of fibrillin-1 in aortic tissue, with no discernible pattern, in contrast to the WT group. In an in vitro study with mgΔ^lpn^ cells, similar alterations were noted^36^, suggesting that the dominant-negative mutation changes the structure of the fibrillin-1 network. This alteration might result in a more severe phenotype, as Ramirez et al.^54^ reported a negative impact on the structural role of aorta tissue when fibrillin-1 was altered.

Interestingly, it is considered that the fibrillin-1 network, microfibrils, and elastic fibers require fibronectin protein, as a prerequisite for assembly^8,47,56,57^. In this study, Fbn1 mgΔ^lpn^ mice aorta tissue showed a significant reduction in fibronectin compared to the WT group, suggesting a loss of capacity for correct elastic fiber assembly in Fbn1 mgΔ^lpn^ mice aorta tissue, which might contribute to the elastic fiber fragmentation observed.

Volume electron microscopy 3D-ultrastructural analysis of the IEL showed a clear loss of integrity of its normal fenestrated sheet like structure. Instead of a sheet of interconnecting elastic fibers only fragments were observed in the Fbn1 mgΔ^lpn^ mice group. It is therefore likely that fibrillin-1 is a key component of its structure the loss of integrity of the IEL sheet could explain the partial loss of adhesion of the endothelium to the elastic lamellae of the internal intima. Indeed the loss of integrity of the endothelium in the Fbn1 mgΔ^lpn^ mice aorta was a key finding of this study.

Endothelial dysfunction has been observed in MFS patients and animal model^58–61^, making it a therapeutic target^62,63^. Fibrillin-1 is understood as a crucial protein for endothelium adhesion through integrin binding^40,64^. Interestingly we identified a significant decrease in integrin α5β1 and additionally, also observed a significant reduction in perlecan and collagen type IV in the Fbn1 mgΔ^lpn^ mice group, both crucial components of the basement membrane (BM)^23^. We hypothesis that the reduction of fibrillin-1 expression and the loss of integrity of the IEL brings about complex multifactorial changes to fibrillin-1 associated proteins involved in cell adhesion and leads to the endothelial dysfunction observed in MFS.

Furthermore, we consider these changes could affect tunica intima, basement membrane, and endothelium adhesion, which could result in an atherosclerosis process, although this was not observed in the current study. This absence of atherosclerosis might be explained by a reduction of integrins and fibronectin, as these components are known to be enhanced in the atherosclerosis process^65^. However, the fragility of the tunica intima, basement membrane, and endothelium adhesion could also be associated with aortic dissection, explaining its prevalence in MFS patients^2,20^, as well as in the mgΔ^lpn^ model^38^.

The reduction in the integrity of elastic fibers in the tunica media of MFS aorta tissue might be a consequence of the reduction in fibrillin-1, as well as mentioned, a strong positive correlation between fibrillin-1 signal intensity and elastic fiber integrity (Fig. 1E). This hypothesis is supported by the understanding that fibrillin-1 microfibrils are associated with elastogenesis, facilitating the formation of elastic fibers from elastin polymers^7,39,66^.

Additionally, the reduction of fibronectin could be associated with the diminished integrity of elastic fibers, as fibronectin is considered a critical prerequisite for the fibrillin-1 network and elastic fiber assembly^8,48^. The compromised integrity of elastic fibers could be linked to the reduction of integrin β1, which is associated with elastic fiber assembly^8^, or the disconnection of elastic fibers on the cell surface of VSMC, which require integrins for adhesion^2^.

In addition to alterations in the elastic fiber structure, we observed a significant increase in collagen fiber deposition in the tunica media of the MFS group, primarily at the sites of elastic fiber rupture, as detected by SHG. Although SHG does not differentiate between types of collagens, this technique provides precise information on collagen distribution^67–71^, as demonstrated in other MFS model studies^72–74^.

Collagen type III has been associated with the interface between elastic fibers and the VSMC network in physiological conditions^75,76^. In this study, a significant reduction in collagen type III was observed, and in regions where elastic fiber fragmentation was present, the absence of collagen type III was noted. D’hondt et al.^77^ provided an animal model (Ehlers-Danlos syndrome model) demonstrating a loss of collagen type III and a significant intercellular space between VSMC and elastic fiber in aorta tissue, highlighting the significance of type III collagen fibrils in preserving the vessel wall’s ECM architecture.

This study observed a significant reduction in critical ECM components in the aorta tissue of the Fbn1 mgΔ^lpn^ mice group. However, a substantial increase in collagen type I was also observed, similar to other MFS mouse models^78^ and the tunica media of MFS patients^79^. In physiological conditions, collagen type I is involved in providing support to the aorta ECM^80^. However, high expression is implicated in aortic dissection, medionecrosis, and atherosclerosis in the aorta^81^. Additionally, the Fbn1 mgΔ^lpn^ mice group exhibited a disorganized collagen fiber network similar to that observed in MFS patients^81^.

Collagen type III is associated with tissues that must withstand stretching^82^, the significant reduction in collagen type III and the increase in collagen type I in Fbn1 mgΔ^lpn^ mice could therefore contribute to aorta stiffening.

Increased deposits of collagen have been associated with elevated vascular smooth muscle cells (VSMC)^78,79^. This study observed a significant increase in α-actin smooth muscle in the MFS tunica media, indicating a possible association with an unbalanced ECM in the Fbn1 mgΔ^lpn^ mice group. Additionally, alterations in VSMC can change the contractile properties in the aorta and are associated with the genesis of aortic aneurysms^79,83^.

The alteration of aorta ECM in the Fbn1 mgΔ^lpn^ mice group can lead to stiffness and reduced aorta elasticity, factors associated with aortic alterations^80^. Although biomechanical properties of the aortic wall were not directly assessed in the present study, previous investigations using various Marfan syndrome mouse models have demonstrated that elastic fiber fragmentation and increased collagen deposition contribute to circumferential stiffening of the aorta. This stiffening progresses in parallel with the degree of aortic dilatation^84^, leading to reduced energy storage capacity and impaired cyclic distensibility of aortic tissues^85^. Furthermore, these structural changes result in elevated aortic pulse wave velocity, a well-established surrogate marker of aortic stiffness^86^. Importantly, Tarraf et al.^85^ reported that aortic stiffening in the Fbn1 mgΔ^lpn^ mouse model correlates strongly with the extent of aortic dilatation.

The alteration of the ECM in Fbn1 mgΔ^lpn^ mice possibly contributes to disturbed aorta blood flow. de Souza et al.^38^ observed alterations in ECM and the pattern of aorta blood flow in mgΔ^lpn^ mice with and without descending thoracic aortic aneurysm and/or dissection (dTAAD). Interestingly, only animals with significantly reduced aortic blood flow were associated with both dTAAD and spinal deformities. In the Fbn1 mgΔ^lpn^ mice group, we observed a marked reduction in abdominal aortic blood flow as well as a severe spinal curvature when compared to WT controls (WT: 5.98 ± 1.09 vs. Fbn1 mgΔ^lpn^ mice: 3.87 ± 0.70; Supplementary Fig. 1C, D), Although no histological or macroscopic evidence of dissection was identified in the thoracic aorta segment analyzed (TIII–TVIII), Fbn1 mgΔ^lpn^ mice showed significant dilatation of the aortic root and ascending aorta (Table 1, Supplementary Fig. 2).

In addition, most cardiac parameters, including cardiac output and chamber volume, remained unchanged, suggesting that the reduced abdominal aortic blood flow does not result from impaired systolic function or valvular defects. We propose that elastic fiber fragmentation and aortic dilation alter systemic blood distribution, redirecting flow toward the more compliant vasculature of the upper body and thereby reducing distal perfusion. This interpretation is supported by the significant correlation between blood flow and elastic fiber integrity. Similar flow disturbances have been associated with aortic dilation in MFS patients^87–90^, consistent with our findings in the aortic root and ascending aorta (Table 1).

This study demonstrates a in the Fbn1 mgΔ^lpn^ mice significant structural disorganization of the aortic wall, including reduced fibrillin-1, fibronectin, and integrin expression, associated with elastic fiber fragmentation, disruption of the internal elastic lamina, and partial endothelial detachment. Besides, ECM alterations in the tunica media included reduced collagen type III and increased collagen type I, suggesting a remodeling process and loss of elasticity. These changes were accompanied by abnormal blood flow, aortic dilation, and early diastolic dysfunction. Together, our findings highlight critical ECM remodeling and compromised structural integrity in the MFS aorta that may underline the pathogenesis of vascular complications, which can contribute to understanding the pathophysiology of the aorta’s wall structure in MFS and may also contribute to the clinical management of the disease.

## Methods

### Animals

This study used twenty 6-month-old male mice of the C57Bl/6 strain. Thus, 10 animals were wild-type (WT) and 10 were heterozygotes from the mgΔ^lpn^ model (Fbn1 mgΔ^lpn^ mice)^36^ (both from Instituto de Biociências at the Universidade de São Paulo, São Paulo, Brazil), and had body weight of 30.06 g ± 3.12 g, and 30.15 g ± 2.45 g; WT and Fbn1 mgΔ^lpn^ mice groups respectively. mgΔ^lpn^ model exhibited aneurysm, aortic dissection, and classic phenotype (alteration of eye, bone, and cardiovascular) of MFS disease^36–38^. The study was approved by the Institutional Animal Care and Use Committee of the Instituto de Biociências at the Universidade de São Paulo. Protocol ID: CEA/IBUSP 272/2016 Process 16.1.632.41.7; we confirm that all methods were performed in accordance with the ARRIVE guidelines.

### Morphological and morphometric study

Fragments of thoracic aorta samples from the third to the eighth thoracic vertebrae (T_III_-T_VIII_ region) were collected, fixed in 4% paraformaldehyde in 0.1 M PBS (pH 7.4), and embedded in resin (Technovit Kit 7100, Kulzer, Hanau, Germany). The Technovit Kit 7100 embedded methods preserve tissue structure better than other methods. Five slides of the thoracic aorta from each animal were cut with 4-micron-thick transverse slices and stained with Toluidine Blue. For the examination of the samples, we used the A Carl-Zeiss Axio Scope Microscope A1.

The thoracic aorta sample was imaged at 400x and 1000x magnification. For analysis of the elastic fibers, integrity was measured following the protocol used by Gerdes Gyuricza et al.^91^. For analysis of the endothelium, we counted the number of endothelial cells per unit area for the typical squamous endothelial cells and also for endothelium where cells were partially detached, using the “point” tool of the ZEN software (Carl Zeiss Microscopy GmbH, Jena, Germany). After this, we divided the number of endothelial cells by the area of the tunica intima, which was measured by the “contours” tool of the ZEN software and was calculated as follows:

$$\:Endothelium\:attached\:Index=\frac{Number\:of\:\:endothelium\:cells\:\:\:}{area\:of\:the\:tunica\:intima}$$

and

$$\:Endothelium\:detached\:Index=\frac{Number\:of\:partially\:detached\:endothelium\:cells\:\:\:\:}{area\:of\:the\:tunica\:intima}.$$

### Electron microscopy ultrastructure analysis

Samples were prepared for serial block-face scanning electron microscopy (SBF-SEM) and transmission electron microscopy (TEM) using a staining method for elastic fibers developed by Lewis et al.^92^. Three 1mm^3^ samples of the aorta from each of three animals were fixed in modified Karnovsky’s fixative (2.5% glutaraldehyde and 2% paraformaldehyde in 0.1 M cacodylate buffer at pH 7.2) for 2 h. After fixation, samples were washed in sodium cacodylate buffer 3 times for 10 min and distilled water for 5 min. The samples were post-fixed in 1% osmium tetroxide for 1 h and washed with distilled water three times for 20 min. Subsequently, samples were put in 0.5% filtered tannic acid in distilled water for 2 h, after which, samples were washed with distilled water three times over 30 min and left overnight in 2% aqueous uranyl acetate. Samples were then dehydrated in a 70–100% ethanol series. They were stained with 1% uranyl acetate for 2 h followed by lead acetate in 1:1 ethanol and acetone and then placed into a 1:1 acetone and araldite resin mix (Araldite monomer CY212 and DDSA hardener) for 1 h. BDMA accelerator was added to the pre-made Araldite resin making continuous resin changes to samples, 6 times for 2 h each change. Samples were embedded in flat embedding molds and the blocks polymerized at 60 °C for 48 h. Finally, the polymerized blocks were trimmed and mounted on Gatan 3view pins for either SBF-SEM.

### Serial block face scanning electron microscopy (SBF-SEM)

Samples were examined using a Zeiss Sigma VP field emission gun scanning electron microscope (Carl Zeiss Meditec, Jena, Germany) equipped with a Gatan 3View2 system, where data sets of up to 1000 4 K x 4 K images were acquired every 75 nm at a pixel resolution of 7.3 nm. Three-dimensional (3D) reconstructions of data sets were created with Amira 6.0.1 software, using manual segmentation similarly to Feneck et al.^93^, and Souza et al.^94^.

### Immunofluorescence

Fragments of thoracic aorta samples from the third to the eighth thoracic vertebrae (T_III_–T_VIII_ region) were fixed in 4% paraformaldehyde in PBS. After this procedure the samples were embedded in paraffin. Paraffin Section (7 μm thick) were collected on SuperFrost Plus slides (Thermo Fisher Scientific, Inc., Waltham, MA). Samples were dewaxed with xylols 3 times for 15 min at 56 °C and rehydrated through graded ethanol. For fibrillin-1, fibronectin, collagens type I and type III, alpha-smooth-muscle, and integrin α_5_β_1_ the antigen retrieval was performed by heating the slides in citrate-EDTA buffer (10 mM citric acid, 2 mM EDTA, 0.05% Tween-20, pH 6.2) in a microwave oven three times for 1.5 min each at 50% power, as described by Benne et al.^95^. Slides were washed twice for 2 min each in PBST (PBS containing 0.1% Tween-20) and for 5 min in PBS. Slides were incubated with blocking solution (10% normal bovine serum [NGS] in PBS) at room temperature for 1 h and then with anti-fibrillin-1 [mrFbn1-C-74-F (provided by Dr. Dieter Reinhardt, McGill University, Montreal, Canada) – 1:500] antisera^96^, anti-fibronectin [Abcam, Cambridge, United Kingdom, ref. 45688–1:500], anti-collagen type I [Abcam, Cambridge, United Kingdom, ref. 6308–1:1000], anti-collagen type III [Abcam, Cambridge, United Kingdom, ref. 7778–1:800], anti-alpha-smooth-muscle [Abcam, Cambridge, United Kingdom, ref. 124964–1:500], anti-integrin α5 (Abcam, Cambridge, United Kingdom, ref 150361–1:300), and anti-integrin β1 (Abcam, Cambridge, United Kingdom, ref 30394–1:300) antibodies diluted in blocking solution overnight at 4 °C.

For the analysis of perlecan and collagen type IV, antigen retrieval was performed by transferring the slides to a hot citrate-EDTA buffer solution (10 mM citric acid, 2 mM EDTA, 0.05% Tween-20, pH 6.2). The slides were incubated in this solution at 98 °C for 20 min in a water bath. Following this step, the solution and slides were allowed to cool down for 20 min at room temperature. Once cooled, the slides were removed from the container and rinsed three times with distilled water (5 min per wash). Subsequently, the tissue underwent antigen retrieval using Proteinase K (brand: [insert brand name]), diluted with PBS in equal parts. The slides were incubated with this solution for 5 min at room temperature. Afterward, the excess liquid was removed, and the slides were washed three times with TBS (5 min per wash).The slides were incubated with a blocking solution (10% normal bovine serum [NGS] in PBS) at room temperature for 1 h. This was followed by overnight incubation at 4 °C with the following primary antibodies, diluted in the blocking solution: anti-perlecan (Thermo Fisher Scientific, Waltham, Massachusetts, United States), ref. MA1-06821, 1:500) and anti-collagen type IV (Abcam, Cambridge, United Kingdom, ref. 6586, 1:500).

The sections were washed with PBST three times for 5 min each and incubated for 1 h at room temperature with secondary antibody in blocking solution. Sections were washed three times for 10 min each in PBST and mounted in ProLong Gold Anti-Fading Reagent with 4′,6-diamidino-2-phenylindole (DAPI; Invitrogen, Carlsbad, California, United States), viewed and photographed using a Carl-Zeiss Imager.D2 microscope. The intensities of the color staining were analyzed with the “rectangle profile” tool of the Zen software. Intensity values are expressed as intensity per pixel (intensity/px), and area measurements are expressed in square pixels (px²), as provided by the software’s quantification tools.

### Immunomarker for elastin and second-harmonic generation (SHG)

Fragments of the thoracic aorta were fixed in 4% paraformaldehyde in 0.1 M sodium PBS (pH 7.4), and embedded in paraffin. Five micrometer-thick transversal slices were performed. All samples were stored in an oven at 60 °C for 30 min, after which they were dewaxed two times with Roti^®^Histol (Carl Roti, Karlsruhe, Germany), ref. Art-Nr 6640, Germany) for 6 min each. Subsequently, the samples were emersed sequentially in isopropanol, ethanol 96%, ethanol 70%, and water for 6 min in each solution. Antigen retrieval was performed by heating the slides in citrate buffer pH 6.0 (Sigma-Aldrich, St. Louis, MO, USA ref. C9999) in a microwave for 15 min at 180 watts. After the samples had cooled to room temperature, they were washed three times in PBS for 5 min each. Slides were incubated with blocking solution (5% normal donkey serum [NDS] (Abcam, Cambridge, United Kingdom, ref. ab7475), in PBS) at room temperature for 1 h, and then with anti-tropoelastin (Abcam, Cambridge, United Kingdom, ref. AB21600) diluted in blocking solution (1:100) overnight at 4 °C. Sections were washed with PBS three times for 5 min each, incubated for 1 h at room temperature with a secondary antibody (Invitrogen, California, United States, ref. 21443) in blocking solution (1:1000), and then washed three times for 10 min each in PBS. A Leica SP8-MP multiphoton confocal microscope was used to analyze collagen deposition by second-harmonic generation (SHG). The same section was used for both Immunomarker (tropoelastin) and SHG (collagen fibers) imaging using a tunable Chameleon Ultra II laser at 880 nm with signal collection in the forward direction. Images were taken at 3× magnification using the objective HCXIRAPOL 25 × 10.95, were processed using FIJI software (National Institutes of Health, Bethesda, MD, USA), and then the intensity of SHG signal was quantified using the “rectangle profile” tool of the Zen software. To measure the intensity of collagen in tunica media, the tunica media was isolated using the Photoshop CS6 software and then quantified using the “rectangle profile” tool of the Zen software. Intensity values are expressed as intensity per pixel (intensity/px), and area measurements are expressed in square pixels (px²), as provided by the software’s quantification tools. Collagen fiber diameters were measured using FIJI software (National Institutes of Health, Bethesda, MD, USA). SHG images were acquired with a 25× magnification objective (Leica HCX IRAPO L 25×/0.95 WATER), using 3× digital zoom and a resolution of 1024 × 1024 pixels.

In the tunica adventitia, collagen fibers were visually identified and individually measured using the line tool. For each image, ten distinguishable fibers were selected, and three measurements were taken at different positions along each fiber to calculate an average diameter. In the tunica media, collagen fibers were identified using the threshold tool in FIJI, following the method described by Chen et al.^53^. Each image was divided into three regions (as illustrated in the Supplementary Fig. 3), and within each region, all visible collagen fibers were measured using the Straight tool. When possible, three equidistance points were selected along each fiber to obtain a representative thickness. In cases where fibers could not be reliably measured due to overlapping structures or poor signal, they were excluded from the analysis.

### Aorta blood flow

All animals were anesthetized with general anesthesia (0.01/100 mg Ketamine^®^ and Xylazine^®^ (4:1)) by the intraperitoneal route. After this procedure the abdominal aorta was dissected above the infra-phrenic artery near the aortic hiatus, after which an ultrasound flow-probe 2SB/T206 (Transonic Systems Inc, Ithaca, NY) was placed around the vessels. The microsurgery procedures were performed under a surgical microscope M900 D.F. Vasconcellos 1.2.6.

### Echocardiography

Echocardiographic assessments were conducted on ten 6-month-old male C57BL/6 mice, comprising five wild-type (WT) animals and five Fbn1 mgΔ^lpn^ mutant mice. All animals were anesthetized using 2% isoflurane, and imaging was performed with a Vevo 2100 high-resolution ultrasound system (VisualSonics, Toronto, Canada) equipped with a 40 MHz transducer. Measurements were acquired in accordance with the guidelines of the American Society of Echocardiography.Two-dimensional images of the aortic root and ascending aorta were obtained from the suprasternal view in the longitudinal plane. Cardiac function was evaluated using the following parameters: Myocardial Performance Index, A′ wave, E′ wave, S′ wave, Isovolumetric Contraction Time (ICT), Isovolumetric Relaxation Time (IRT), A wave, E wave deceleration time, Ejection Time (ET), A′/E′ ratio, E′/A′ ratio, E/A ratio, E/E′ ratio, Ejection Fraction (EF), and Shortening Fraction (SF). All measurements were averaged over three representative cardiac cycles per animal. Echocardiographic analyses were performed in a blinded manner to ensure unbiased interpretation.

### Thoracic spine analysis

Before sacrifice for collection of tissue samples, mice were anesthetized as described for “Aorta Blood Flow” above. The animals were fixed in the lateral decubitus position with the aid of adhesive tape. Digital radiographic images were obtained with the In-vivo Imaging System FX PRO (Carestream Molecular Imaging, Carestream Health Inc., Rochester, NY, USA). The Kyphosis Index Ratio (KI) was used to assess the severity of the thoracic vertebra deformation, as described by Laws and Hoey^97^.

### Statistical analysis

The Shapiro-Wilk normality test was used to examine the results. A two-sample t-test was used if the data had a normal distribution. A Mann-Whitney U test was employed if the data did not have a normal distribution. In addition, Pearson’s correlation was applied to assess the relationship between fibrillin-1 signal intensity and the Elastic Fiber Integrity (EFI) score, while Spearman’s correlation was used to evaluate the association between aortic blood flow and EFI. All statistical analyses were performed in the GraphPad Prism 5.0. Differences were considered statistically significant at a ρ-value < 0.05.

## Electronic supplementary material

Below is the link to the electronic supplementary material.

Supplementary Material 1

Supplementary Material 2

Supplementary Material 3

Supplementary Material 4

Supplementary Material 5

Supplementary Material 6

Supplementary Material 7

## Data Availability

All data generated or analysed during this study are included in this published article [and its supplementary information files].
