# Supplementary figures and images for: Exploring thoracic aorta ECM alterations in Marfan syndrome: insights into aorta wall structure
Source: Sci Rep. 2025 Jul 22;15:26665. doi: 10.1038/s41598-025-09665-w (PMC12284111; doi:10.1038/s41598-025-09665-w)

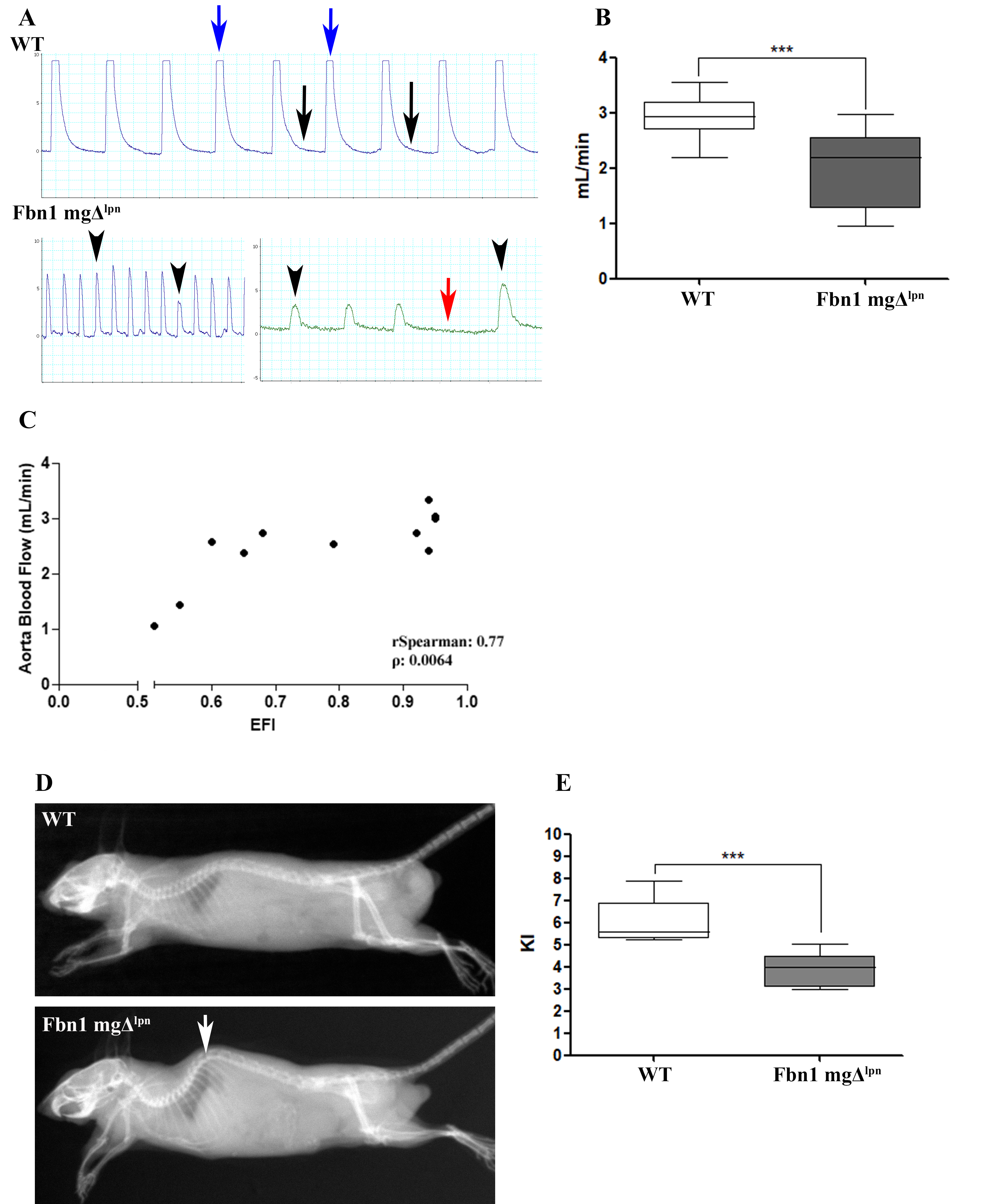

Supplement: Supplementary file 1 — Supplementary Material 1 [file 41598_2025_9665_MOESM1_ESM.tif]

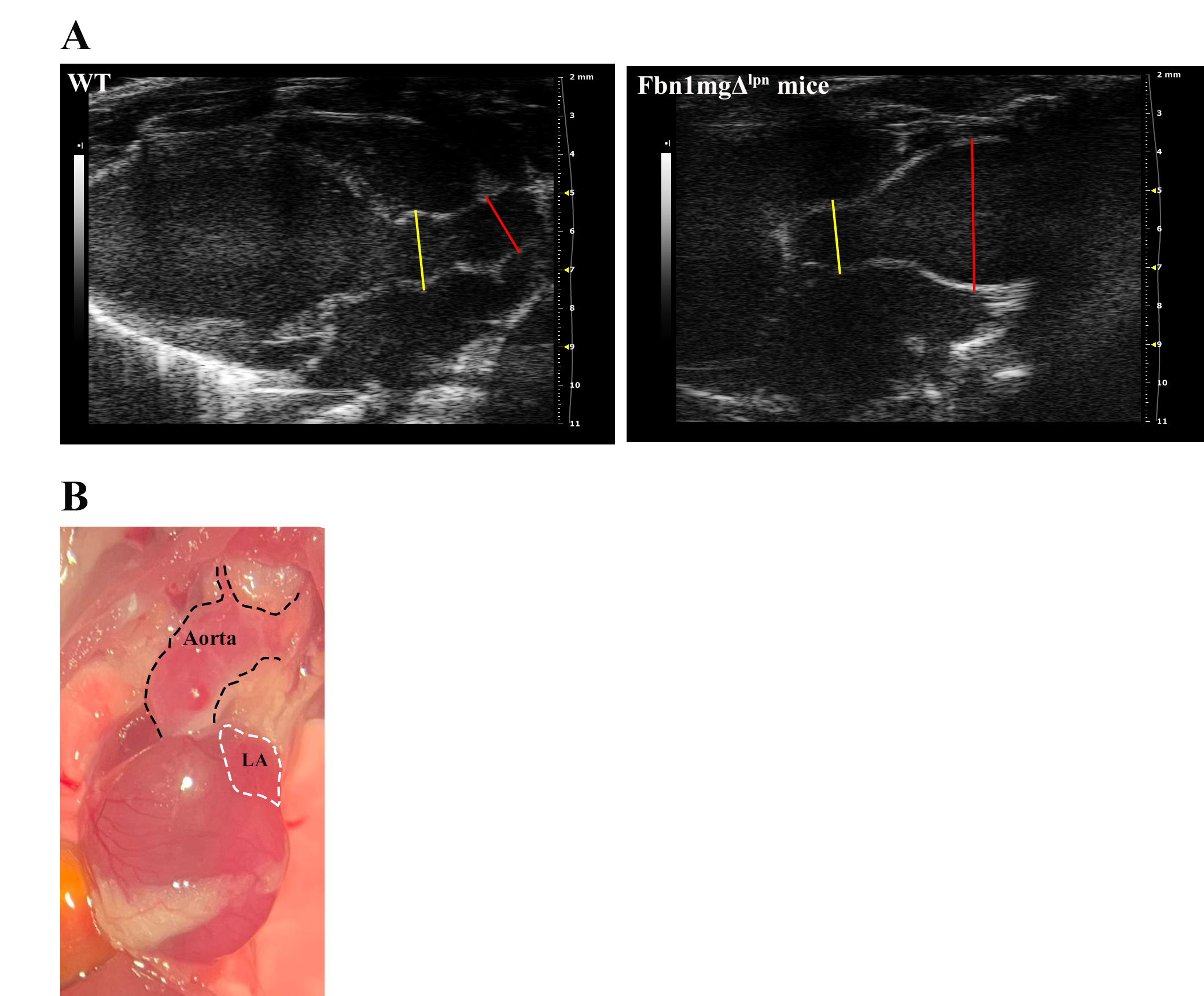

Supplement: Supplementary file 2 — Supplementary Material 2 [file 41598_2025_9665_MOESM2_ESM.tif]

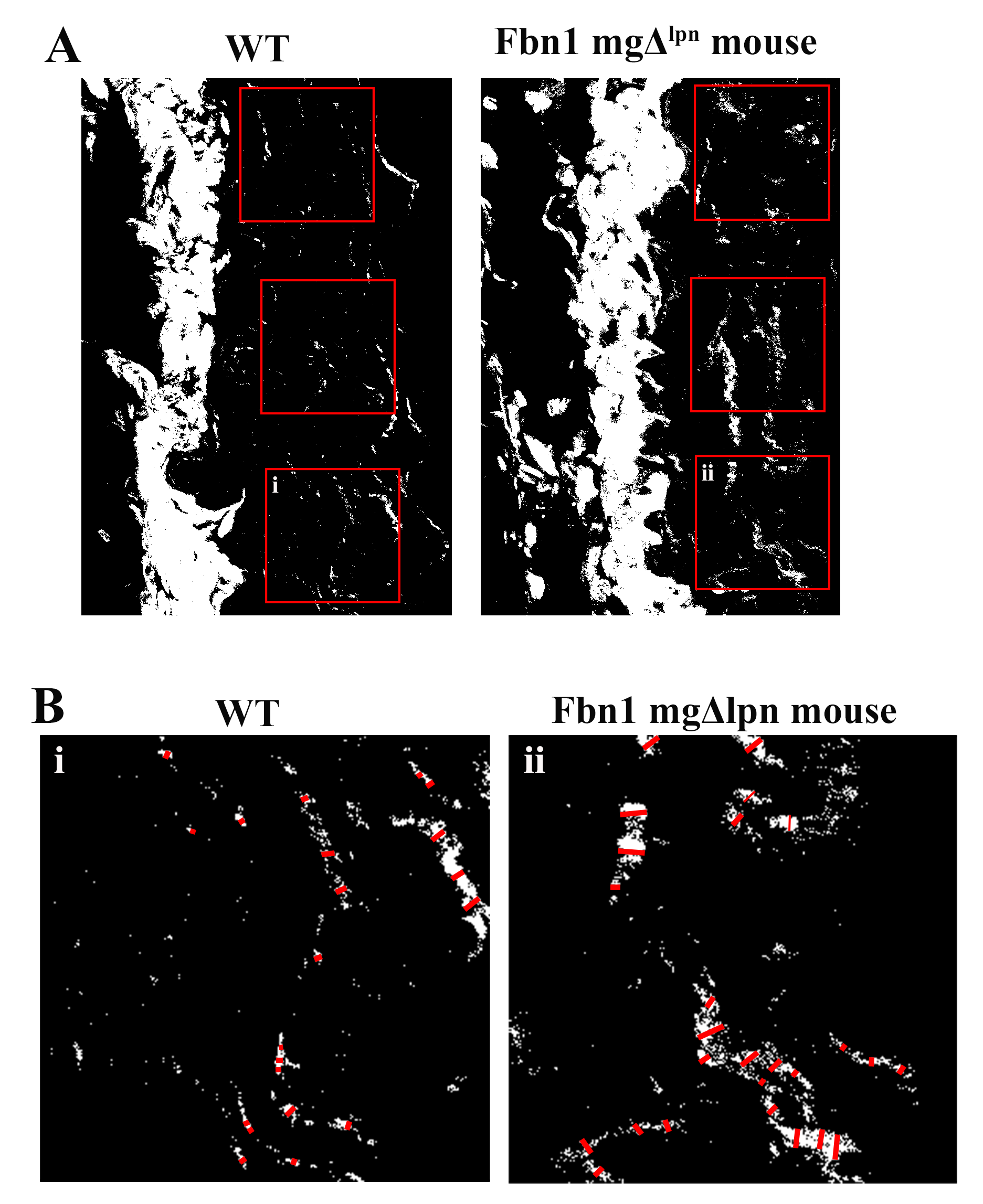

Supplement: Supplementary file 3 — Supplementary Material 3 [file 41598_2025_9665_MOESM3_ESM.tif]
