# Supplementary material for: Exploring thoracic aorta ECM alterations in Marfan syndrome: insights into aorta wall structure
Source: Sci Rep. 2025 Jul 22;15:26665. doi: 10.1038/s41598-025-09665-w (PMC12284111; doi:10.1038/s41598-025-09665-w)
Supplement: Supplementary file 6 — Supplementary Material 6 [file 41598_2025_9665_MOESM6_ESM.pdf]

Supplementary Legends

Supplementary Figure 1. Aortic blood flow spectral curve (A). WT animals present uniform monophasic flow patterns with similar peak systolic velocities (PSVs) (blue arrows) and no alterations in relaxation curves (black arrows). In Fbn1 mgΔ^{lpn} mice animals, the monophasic flow showed varying PSVs (black arrowheads), and a broadened waveform in diastole (red arrow). (B) The Fbn1 mgΔ^{lpn} mice group demonstrated a significant reduction in blood flow (WT 2.93mL/min ± 0.34mL/min, Fbn1 mgΔ^{lpn} mice 1.99mL/min ± 0.63mL/min). (C) Blood flow was also positively correlated with elastic fiber integrity (Spearman r = 0.77, p = 0.0064). (D) Representative X-ray images of WT and Fbn1 mgΔ^{lpn} mice : the Fbn1 mgΔ^{lpn} mice group exhibited spine defect (white arrow). (E) The Kyphosis Index Ratios indicated a severe spine defect (WT 5.98 ± 1.09, MFS 3.87 ± 0.70). (***) ρ <0.001 (Aortic blood flow analysis, and KI: WT n=10; Fbn1 mgΔ^{lpn} mice n=10).

Supplementary Figure 2. Echocardiographic and macroscopic aspects of the aortic root and ascending aorta. (A) Echocardiographic images showing the aortic root (yellow line) and ascending aorta (red line). Aortic dilation of the ascending segment is evident in Fbn1 mgΔlpn mice. (B) Macroscopic view of the heart and aorta. The dotted white line delineates the left atrium (LA), and the dotted black line outlines the aorta. (WT, n = 5; Fbn1 mgΔlpn mice, n = 5).

Supplementary Figure 3. Procedure for measuring collagen fiber thickness. (A) SHG images processed using the "Threshold" tool in FIJI software. In each image, three regions were selected for analysis, one central and two peripherals (highlighted by red squares). (B) Magnified views of the selected regions (i and ii). Red lines indicate the measurements performed using the "Straight" tool in FIJI software.

Supplementary video 1. Illustrates the complex 3D arrangement of the tunica intima, and internal elastic lamina (light blue) in the WT.

Supplementary video 2. Illustrates the complex 3D arrangement of the tunica intima, and internal elastic lamina (light blue) in the MFS.
