# Supplementary material for: Exploring thoracic aorta ECM alterations in Marfan syndrome: insights into aorta wall structure
Source: Sci Rep. 2025 Jul 22;15:26665. doi: 10.1038/s41598-025-09665-w (PMC12284111; doi:10.1038/s41598-025-09665-w)
Supplement: Supplementary file 7 — Supplementary Material 7 [file 41598_2025_9665_MOESM7_ESM.pdf]

Manuscript ID no: 61f961a9-d6fe-489b-a8ec-1f07592c9ab4 v2.0

Title: Exploring Thoracic Aorta ECM Alterationsin Marfan Syndrome: Insightsinto Aorta Wall Structure

Elastic fibers integrity index (EFI)

WT	Fbn1 $mg\Delta^{lpn}$
1,000	0,562500
1,000	0,562500
0,750	0,750000
1,000	0,437500
0,880	0,6666667
1,000	0,5416667
0,830	0,562500
0,880	0,750000
1,000	0,4666667
1,000	0,5416667
1,000	0,7083333
0,750	0,875000
1,000	0,8333333
0,875	0,6458333
1,000	0,7083333
0,875	0,3958333
1,000	0,4583333
1,000	0,687500
1,000	0,5833333
0,750	0,5833333
0,875	0,4583333
1,000	0,875000
1,000	0,750000
1,000	0,625000
0,875	0,6166667
0,875	0,6166667

1,000	0,6166667
1,000	0,5666667
	0,5166667
	0,700000
	0,6166667
	0,600000
	0,4833333
	0,6333333
	0,7666667
	0,5666667
	0,6166667
	0,6333333
	0,6333333
	0,7666667
	0,7666667
	0,800000
	0,800000
	0,800000

Fibrillin-1 Intensity

**WT**	**Fbn1 mgΔ^{lpn}**
40,980000	21,511320
58,420000	16,301840
33,750000	20,924290
36,010000	41,199520
29,400000	36,033870
27,801840	16,059750
33,663440	17,796770
28,042650	23,937080
33,374570	22,740550
44,437730	

Correlation between EFI and Intensity of Fibrillin-1

EFI	Intensity of Fibrillin-1
0,94	36,012940
0,95	37,365970
0,86	28,602140
0,90	33,703190
0,90	33,374570
0,65	12,159970
0,71	21,217810
0,51	17,796770
0,60	15,303810

Endothelium attached

WT	Fbn1 mgΔlpn
83,6	78,9
69,2	90,8
58,8	33,0
52,5	25,8
85,6	51,4
47,5	64,1
66,9	37,0
74,0	29,9
57,0	39,6
39,9	14,4
44,5	20,9
62,3	51,1
21,7	40,8
61,8	41,7
53,8	63,2
65,9	53,6
77,7	50,3

45,8	40,9
39,8	57,1
59,9	36,2
48,1	46,2
65,3	58,3
0,6	55,7
38,0	
69,9	

Endothelium partially detached

**WT**	**Fbn1 mgΔlpn**
0,0	3,3
0,0	7,6
0,0	44,0
0,0	23,7
0,0	11,4
7,1	32,1
0,0	31,3
0,0	24,9
0,0	0,0
8,9	48,2
0,0	17,9
0,0	6,8
9,3	13,6
1,7	13,9
8,0	0,0
0,0	14,3
0,0	0,0
0,0	17,5
9,2	6,3
6,3	18,1

0,0	8,4
0,0	6,5
0,4	11,1
5,4	
0,0	

Intensity of Fibrillin-1 in tunica intima

**WT**	**Fbn1 mgΔlpn**
15,423520	9,515936
21,822250	11,393690
11,166430	10,610070
16,161850	5,993671
21,867210	7,015808

Perlecan in tunica intima

**WT**	**Fbn1 mgΔlpn**
43,323090	17,790720
30,516880	16,530990
28,270110	22,445510
18,534270	25,465640
29,852480	13,160810
37,439990	14,886400

Col IV in tunica intima

**WT**	**Fbn1 mgΔlpn**
31,770100	18,278250
43,585480	22,902440
42,128200	23,027980
45,683530	30,821730
30,417950	10,903710

Integrin α5 in tunica intima

WT	Fbn1 mgΔlpn
21,091910	22,400190
16,086120	9,192742
26,411180	21,291200
26,264750	18,631790
27,464690	16,195950
28,906830	22,275440
23,881950	19,479830
22,803440	
24,964630	

Integrin β1 in tunica intima

WT	Fbn1 mgΔlpn
11,890370	9,808948
20,650000	6,833106
13,126740	26,480350
22,932030	16,160080
28,853550	10,784300
27,802110	7,775606
14,115490	7,551085
11,643850	
11,178700	

Intensity of fibronectin in tunica media

WT	Fbn1 mgΔlpn
50,442	39,381110
86,742	32,418790
56,141	33,816910
54,421	42,401570
61,822	34,000000

Intensity of α5 in tunica media

WT	Fbn1 mgΔlpn
58,471250	76,503070
44,290600	62,706940
46,943550	69,320200
66,371120	74,954840
105,901200	86,344390
49,383030	69,598180
	113,571000
	98,097040

Intensity of β1 in tunica media

WT	Fbn1 mgΔlpn
53,187660	72,917530
78,460530	58,242390
76,915420	60,202370
90,341440	40,309720
110,274800	87,208700
136,577500	34,278480
62,017510	52,740700

Intensity of α-smooth muscle actin

WT	Fbn1 mgΔlpn
29,910220	73,793500
35,103230	71,672450
57,232050	85,994290
34,740650	70,508830
57,851460	77,878460

Intensity of SHG Total collagen

WT	Fbn1 mgΔlpn

7,552434	9,083968
4,245271	7,965031
7,835066	6,932826
4,178880	5,578712
6,914050	7,405160
6,517457	7,964272
7,542805	5,331428
7,208595	6,847117
6,893279	6,938453
5,606119	6,883757
8,067133	5,877494
6,391609	5,498797
4,590884	9,026243
4,642702	7,389144
4,346549	6,091792
4,412159	7,393737
4,642682	6,629534
4,955372	24,930610
2,696426	3,221369
2,040779	5,343052
14,087640	4,805523
13,242260	13,008790
	17,702990
	16,583800

Intensity of SHG collagen in tunica media

WT	Fbn1 mgΔlpn
0,449000	0,856000
0,554000	1,336000
0,529000	0,5009507
0,640000	0,7026643

0,1674181	0,6536373
0,5531715	0,5058611
0,3664747	0,2695787
0,3660637	0,4732035
0,3074298	0,3565604
0,1246761	0,2450501
0,3359644	0,522543
0,337226	0,2830366
0,5497429	0,7547869
0,4073606	1,545754
0,8498312	0,8861926
0,6456111	0,7120391
0,4583949	0,4324606
0,3211606	0,669401
0,6248032	0,7751215
0,3476241	0,7974296
0,6249051	0,5338892
0,6438766	0,6983703
1,094079	0,6019636
	0,4532203

Thickness of collagen fibers in tunica adventitia

**WT**	**Fbn1 mgΔlpn**
0,605800	0,5721667
0,5482333	0,6019334
0,6148462	0,6509333
0,6382333	0,8170334
0,6118667	0,6996667
0,601900	0,6212667
0,5755128	0,7101667
0,5492333	0,5976667

0,702400	0,7772667
0,588200	0,6310334
0,6618621	0,6745667
0,6576774	0,734200

Thickness of collagen fibers in tunica media

WT	Fbn1 mgΔlpn
0,1963286	0,319500
0,1905286	0,2754286
0,1932208	0,4323809
0,1660893	0,2827533
0,1661837	0,2580779
0,153200	0,2806429

Intensity of collagen type III

WT	Fbn1 mgΔlpn
22,261010	20,888030
41,504440	28,165990
43,839870	31,196410
39,515100	15,452340
36,800000	22,160000

Intensity of collagen type I

WT	Fbn1 mgΔlpn
8,067406	45,582020
27,455320	83,895000
21,645940	129,119200
25,992360	45,111400
31,108450	44,034130
30,185410	27,667520
23,650000	38,889170

	29,470370

Aorta Blood Flow (mL/min)

**WT**	**Fbn1 mgΔlpn**
3,2040	1,4919
3,5058	1,2092
3,5468	1,0025
3,2419	1,8284
3,1879	1,6660
2,6870	2,1600
2,8072	2,2371
2,7894	2,0919
2,6265	2,0795
2,1912	2,0953
2,3577	2,8815
2,6400	2,7672
2,4869	2,5449
3,0459	2,7312
2,7994	2,2423
3,1346	2,5234
2,9237	2,5545
3,0624	2,2923
2,8445	2,2362
2,8885	2,7107
3,2175	2,2223
3,1846	2,5680
3,0375	2,9765
	2,3706
	0,9528
	1,1039
	1,0991

1,0779
1,2317
1,1508
1,2420
1,3679
1,3563
2,6067
2,4200
2,5870

Kyphosis Index

5,571616	4,000000
5,356748	3,000000
5,237189	3,000000
7,899996	3,184589
5,832130	3,971207
5,609398	3,809964
4,514534	4,495715
4,670590	4,429208
4,332657	5,012394
4,129196	3,070105

Correlation between EFI and Aorta Blood Flow (mL/min)

**EFI**	**Aorta Blood Flow (mL/min)**
0,94	3,337280
0,92	2,727525
0,94	2,418950
0,95	2,993200
0,95	3,034520
0,55	1,439600
0,68	2,731200

0,65	2,369740
0,60	2,569620
0,51	1,058425
0,79	2,537900

Echocardiography

	A' (mm/s)	E' (mm/s)	IVCT	IVRT (ms)	MV A (mm/s)	MV Decel (ms)	MV E (mm/s)	MV ET (ms)	MV IVS E' (mm/s)	A'/E'	E'/A'	MV E/A	MV E/E'	TEI	Asc Ao (mm)	Raiz Ao(mm)
WT1	11,229944	29,478604	10,833333	14,166667	197,180160	16,250000	710,182778	59,166667	17,847590	0,380952	2,625000	3,601695	24,091466	0,4225352	1,676418	1,530664
WT2	14,037424	26,470571	21,666667	18,750000	237,284586	26,250000	579,843319	45,416667	18,248651	0,530303	1,885714	2,443662	21,905206	0,8899083	1,740584	1,786011
WT3	11,831541	23,863617	22,500000	16,666667	315,820313	25,833333	608,246528	47,500000	16,644372	0,495798	2,016949	1,925926	25,488447	0,8245614	1,605000	1,695
WT4	18,649721	17,647048	16,333333	16,333333	439,477789	15,666667	579,843319	45,333333	18,649721	1,056818	0,946237	1,319392	32,857808	0,7205882	1,516192	1,530074
WT5	12,433069	11,831469	20,416667	30,833333	322,506523	22,500000	462,872056	54,583333	11,229869	1,050847	0,951613	1,435233	39,122112	0,9389313	1,641567	1,716748
MFS1	18,850263	12,834221	9,166667	17,500000	310,809471	23,333333	364,282068	49,583333	14,839568	1,468750	0,680851	1,172043	28,383652	0,5378151	4,275950	2,37
MFS2	11,631005	11,631005	22,083333	20,833333	290,756966	17,083333	287,414932	52,500000	12,433143	1,000000	1,000000	0,988506	24,711100	0,8174603	3,840735	2,145
MFS3	18,248646	17,446508	14,583333	20,000000	404,385765	18,333333	320,834987	36,250000	18,449181	1,045977	0,956044	0,793388	18,389639	0,954023	3,750982	2,295049
MFS4	11,229946	17,045454	19,166667	31,666667	292,428114	20,833333	232,271473	52,083333	12,633689	0,658824	1,517857	0,794286	13,626594	0,976	3,168564	1,770574
MFS5	24,465220	19,050786	20,000000	20,769231	319,164398	15,769231	310,809309	60,769231	20,855597	1,284211	0,778689	0,973822	16,314776	0,6708861	1,966436	3,3766380
